# Chip Off the Old Block: Generation, Development, and Ancestral Concepts of Heredity

**DOI:** 10.3389/fgene.2022.814436

**Published:** 2022-03-09

**Authors:** Péter Poczai, Jorge A. Santiago-Blay

**Affiliations:** ^1^ Finnish Museum of Natural History, University of Helsinki, Helsinki, Finland; ^2^ Faculty of Biological and Environmental Sciences, University of Helsinki, Helsinki, Finland; ^3^ Institute of Advanced Science Kőszeg (iASK), Kőszeg, Hungary; ^4^ Department of Paleobiology, National Museum of Natural History, Washington, DC, United States; ^5^ The Pennsylvania State University, York, PA, United States

**Keywords:** development, diseases, fertility, genetic force, genetic laws, heredity, inheritance

## Abstract

Heredity is such a fundamental concept that it is hard to imagine a world where the connection between parents and offspring is not understood. Three hundred years ago thinking of the phenomenon of heredity bore on a cluster of distinct philosophical questions inherited from antiquity concerning the nature and origin of substances or beings that lacked biological meaning. We are reminded of this philosophical heritage by the fact that in the 18th century the study of reproduction, embryology and development was referred to as “the science of generation”. It is now clear that reproduction, the biological process by which parents produce offspring, is a fundamental feature of all life on Earth. Heredity, the transmission of traits from parents to offspring via sexual or asexual reproduction, allows differences between individuals to accumulate and evolve through natural selection. Genetics is the study of heredity, and in particular, variation of fundamental units responsible for heredity. Ideas underlying this theory evolved in considerably different and unrelated ways across a number of knowledge domains, including philosophy, medicine, natural history, and breeding. The fusion of these different domains into a single comprehensive theory in 19th century biology was a historically and culturally interdependent process, thus examining genetic prehistory should unravel these entanglements. The major goal of our review is tracing the various threads of thought that gradually converged into our contemporary understanding of heredity.

## Introduction

*How could what is be in the future? How could it come to be?*

*For if it came into being, it is not: nor is it if it is ever going to be in the future.*

*Thus coming to be is extinguished and perishing unheard of.*

Parmenides of Elea (Wedin, 2014)

**FIGURE 1:**
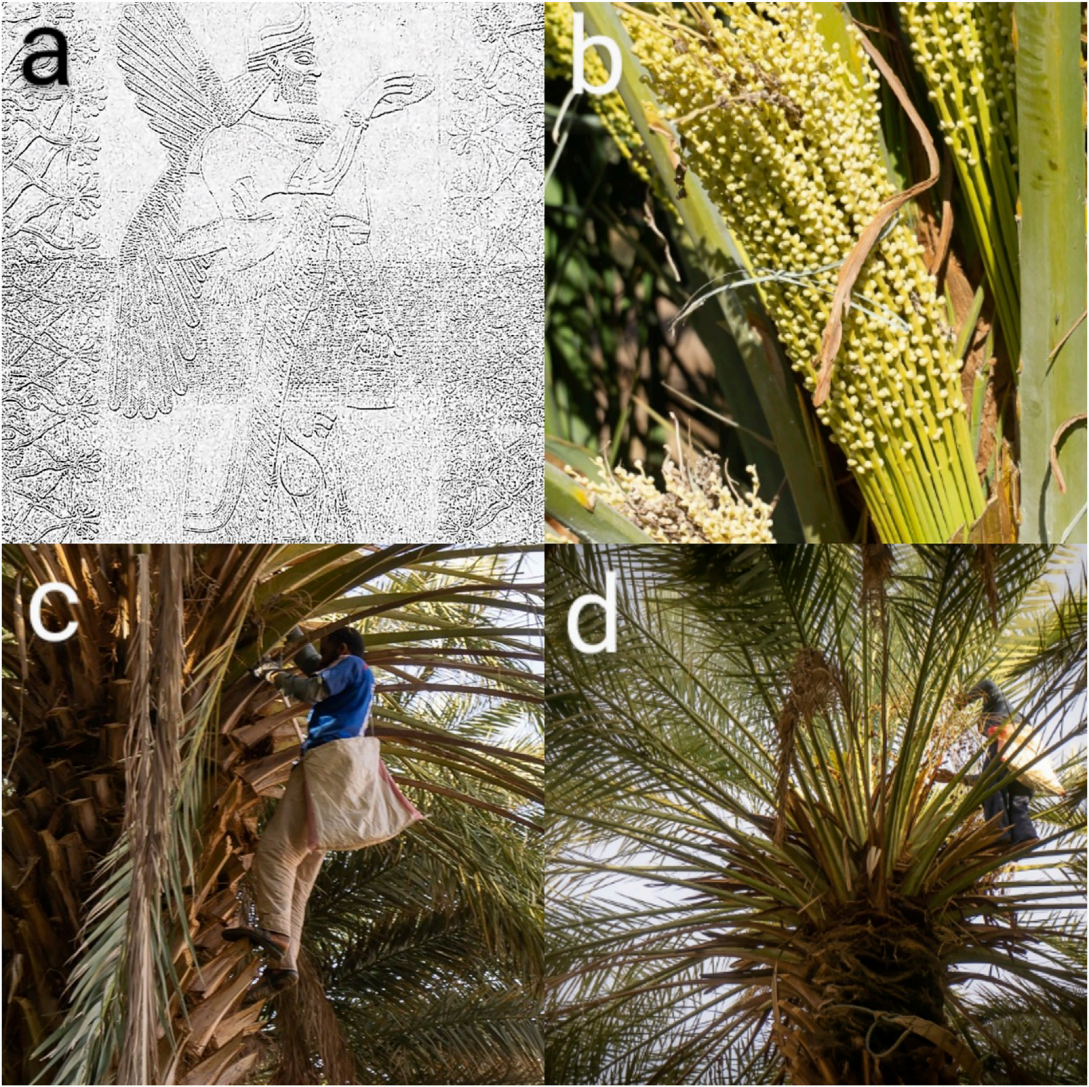
**(A)** Pollen Gods depicted as eagle-headed winged-genies called *Apkallu* with date palm (*Phoenix dactylifera* L.) and pollen bags in their hands. Date palm was regarded as a sacred tree representing life; protective spirits spread the seeds or pollen to create life and fertility. Photo: Assyrian, Nimrud (Kalhu), reign of Ashurnasirpal II (883–859 BCE), alabaster. Middlebury College Museum of Art. **(B)** Date palm is a dioecious species; sexes are borne by separate individuals. The unisexual flowers are pistillate (female) and staminate (male). Male palms generate pollen, whereas female palms yield fruit. Flower stalks are created from the leaf axils in a manner similar to how offshoots are produced. The inflorescence is composed of a long, robust spathe that bursts to reveal several densely packed, thin female branchlets as shown in the photograph. **(C,D)** The two sexes are generated in almost equal quantities in wild palms that reproduce from seed, and this profusion of males provides a significant supply of pollen that, when blown by the wind, pollinates at least enough of the female blooms to perpetuate the species. Understanding this feature was critical for the date palm’s first systematic growers, since by hand-pollination panels **(C,D)** rather than wind-pollination, they could exclude all males but three or four for every hundred females, saving space and work while insuring a greater yield. On the other hand, the separation of the sexes appealed to their religious imagination, and was undoubtedly one among the causes contributing to the palm’s adoration by the early inhabitants of the Tigris-Euphrates area. Photos in courtesy of Dr Vincent Battesti.

**FIGURE 2:**
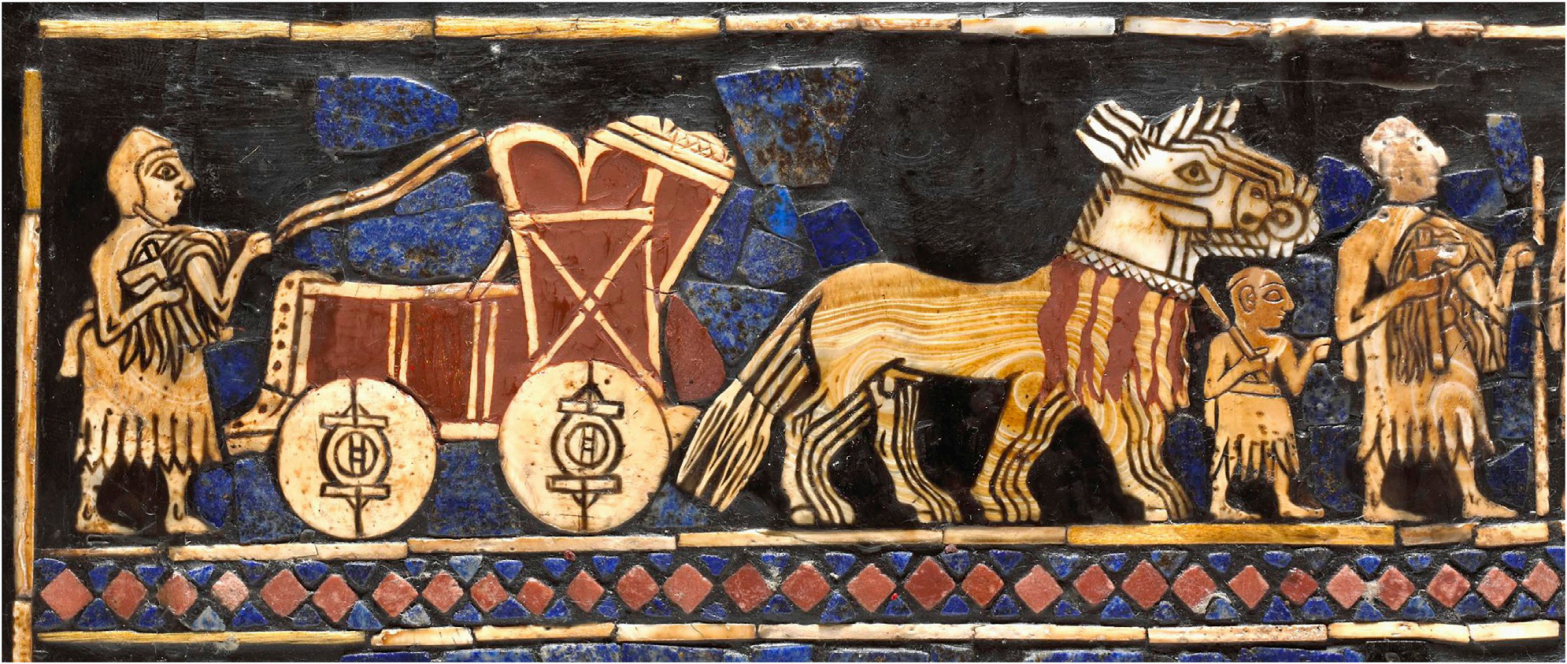
The Standard of Ur with mosaic scenes made from shell, red limestone and lapis lazuli from Early Dynastic III period c. 2500 BCE. The highlighted war scene, depicts the soldiers of the Sumerian army with wheeled wagons pulled by a hybrid kunga. Kungas were described in ancient records as highly desired and expensive creatures, which might be due to the rigorous procedure of breeding them. Each kunga was infertile, just like many hybrid animals such as mules, and they had to be created by mating a female domesticated donkey with a male wild ass, which had to be captured. Wild asses could run faster than donkeys and even kungas and were impossible to tame, making kunga breeding a particularly challenging task. Photo in courtesy of the British Museum (12561001).

Along time ago, humans probably guessed that the birth of a new little animal, which generally looked like its parents, was related to at least one copulation between the parental animals, particularly if the pregnant female had been in estrus at the time of mating. Likewise, some civilizations had notions on plant reproduction as, for example, the ancient Assyrians often depicted a king-like figure the *Apkallu* or winged-genie, apparently pollinating date palms nine centuries before the common era (BCE) (Figure 1) (Zirkle, 1935; Stubbe, 1972; Sturtevant, 2000). Mesopotamians were using hybrids of domesticated donkeys and wild asses for pulling four-wheeled wagons into battle 4,500 years ago—at least 500 years before horses were bred for the same purpose (Bennett et al., 2022). Kungas were the earliest bio-engineered hybrids before the material bases and mechanisms of biological heredity, development, and evolution were explained. Their creation through artificial crossing is a good example of how, without theoretical knowledge, humanity has exploited the seemingly incomprehensive mysteries of heredity on purely empirical basis (Figure 2).

There seems to be a consensus that several factors combined made the conceptualization of hereditary principles difficult (Mayr, 1982; Orel, 1996). The patterns of heredity often do not lend themselves to simple explanations, perhaps leading to a large mythology purporting to explain phenotypic traits in simple Mendelian genetics terms that are perpetuated in biology textbooks.^1^ The appearance of organisms, or phenotype, has a hereditary and environmental component. For instance, some characters are expressed generation after generation (i.e., complete dominance), other traits sometimes skip generations (i.e., recessiveness), some “blend”, like paints (i.e., incomplete dominance), or express themselves simultaneously (i.e., codominance), or are linked to a biological gender (i.e., sex-linked inheritance), others are often inherited together (i.e., genetic linkage), or their interaction affect a single trait (i.e., epistasis) or the proportion of individuals in a population that manifest a condition (i.e., penetrance). See the example in cats listed by Christensen (2000).

The phenotype is also influenced by non-linear interactions between genes and the environment (see Liu et al., 2008; Sa et al., 2016; Pour-Aboughadareh et al., 2019), transgenerational effects, i.e., epigenetics (Bird, 2007; Horvath and Raj, 2008), and by the errors we make in observing, recording, analyzing, and interpreting the data. Beyond the patterns of heredity, how could all the changes an organism experience through its life, or development, be explained? Or, on a much larger time frame, what accounts for adaptations and evolution? In sum, how could all that variation, at times, staggering or seemingly random, be explained with a single theory based on the information available many centuries or millennia ago? Answering these questions has been the subject of deep philosophical mysteries for centuries. For example, Parmenides of Elea c. 475 BCE attempted to explain our inability to understand the underlying unity and fullness of being. He realized that whatever *comes to be* can never really be said to be at all or in other words before all other determinations, the things consist of consisting. Simply said, something metaphysically important takes place here that every philosopher should be aware of.

As Justin E.H. Smith (2006) writes, once the door to *coming-into-being* was opened, and organisms that previously not existed were given the status of full-fledged beings, it is easy to understand why focus quickly shifted to the aspects of biological reproduction. However, as Hopwood et al. (2018) demonstrated, reproduction would include a set of particularly contemporary ideas and practices. Until the end of the 17th century the concept of reproduction itself was lacking from discussions of living organisms (Jacob, 1970). Writers linked the creation of new beings–as we will discuss in our review–to creative processes like brewing, baking, and shaping clay during this time period (see examples in Hopewood, 2018). Prior to the 19th century, most educated people used the term generation to refer to procreation and descent (Stephanson and Wagner, 2015). Minerals and living organisms were similarly included in the “science of generation”, but the human spirit also received particular attention (Hirai, 2011; Wilberding, 2017). The term “reproduction,” which literally means “producing again,” became popular only in the mid-eighteenth century as the ability of all living things–and only theirs–to produce more members of their own species (Hopwood et al., 2018). Writing the history of genetics should also focus on reproduction, whether biologically universal or specific to a certain historical period. Due to the fact that the term “reproduction” only emerged as a unified concept in the late 19th century, the history of the term is fragmented.

The same applies to the discourse of heredity. There is a claim in Jacob’s book “*La logique du vivant* (1970)” that has been validated repeatedly by different biologists and historians: hereditary transmission was not considered as a domain apart from the fluctuations of conception, pregnancy, embryonic development and parturition until the end of the 18th century, and, according to some historians, even until the birth of Mendelism. As Sandler and Sandler (1985) pointed out the concepts of heredity and development were not distinguished clearly as describing distinct phenomena. For the scientists of the 17th and 18th century, heredity constituted only a step within the infinite process of development; it did not occur to them that transmission processes can and should be studied separately. The problem was that, while these scholars did attempt to find links or resemblance between successive generations, whatever they observed seemed to lack consistency. Today, their speculations are an important part of the cross-intellectual debates that have shaped hereditary thinking and eventually forged a scientific discipline from a metaphor.

Herein, we will summarize how the “science of generation,” which included the study of embryonic conception, development, and heredity, has influenced discussions about heredity and how ancient philosophical assumptions about existence, substance, and fertility have influenced empirical research on reproduction and animal breeding in particular. The selected topics are considered important milestones in the early history of heredity. We also address the stronger scientific and philosophical influences of the late 18th and early 19th centuries, up to the work of Imre (Emmerich) Festetics (1764–1847) entitled “About inbreeding (*Ueber Inzucht*)” (Festetics, 1819a; Festetics, 1819b; Festetics, 1819c). This work summarized “The Genetic Laws of Nature (*Die genetischen Gesetze der Natur*)” and reshaped the priorities of theoretical and experimental research later carried out in Brno by Johann Karl Nestler (1829, 1837), Cyrill Franz Napp and several other members of the Moravian Agricultural Society in Central Europe, which eventually led to Gregor Johann Mendel’s experiments on peas (Wood and Orel, 2005; Poczai et al., 2014).^2^ Mendel is, in fact, the internationally best known figure from this conscious and well-organized society. We have chosen Festetics’s work on animal breeding as a convenient landmark to close this narrative, since—around the time of “genetic laws”—the historical paths toward Mendel appear to be more clearly delineable (Wood and Orel, 2001).

## Female Fertility as a Symbol of Heredity

**FIGURE 3:**
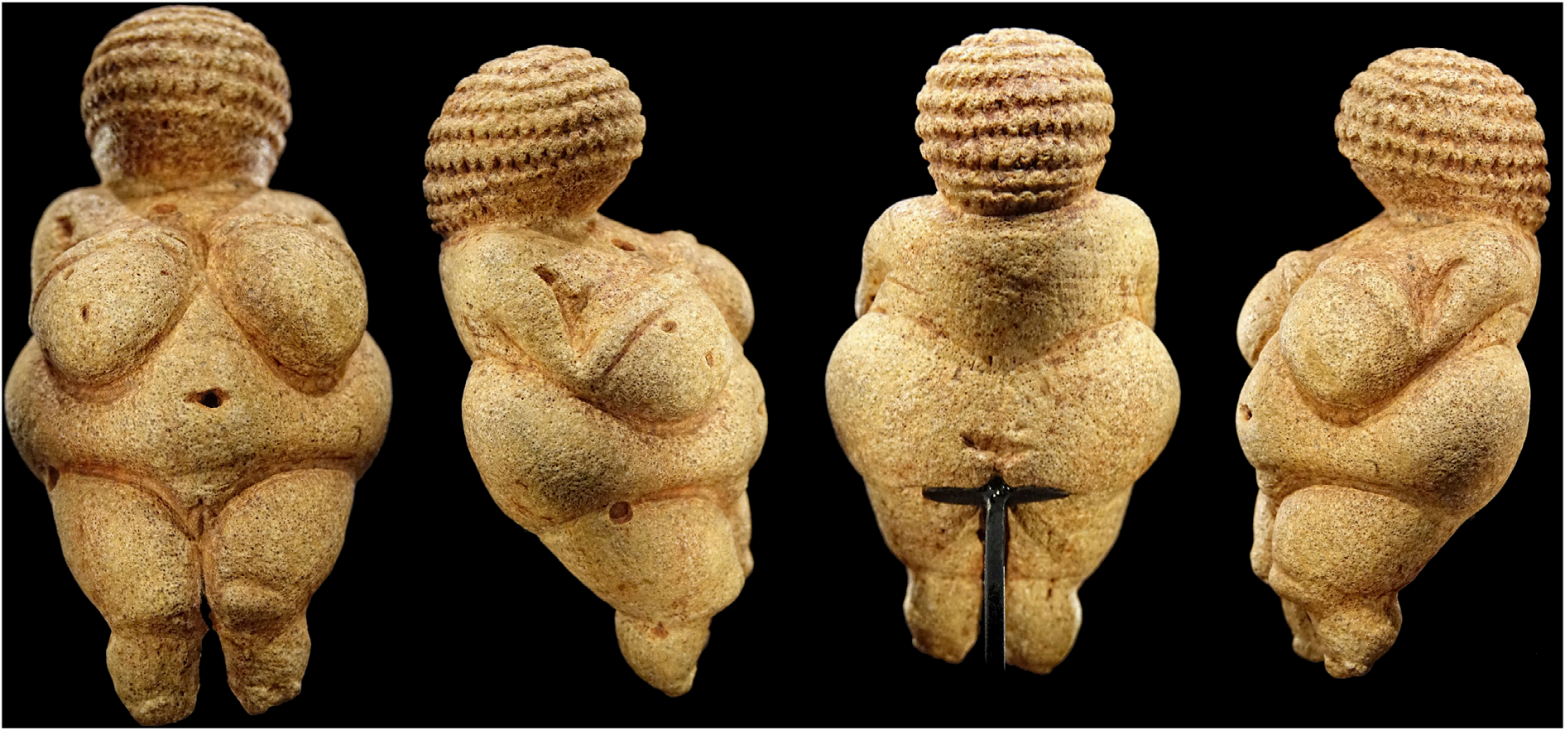
Venus of Willendorf as shown at the Naturhistorisches Museum in Vienna, Austria. Photo: Bjørn Christian Tørrissen (2020).

The ideas we hold about heredity are as old as humankind. Since the birth of human civilization, it is clear that contributions from both sexes are needed to create offspring. However, the degree of their contributions and whether there is a consistent relationship between parents and offspring was not always evident. The earliest artifacts of human figurative art depict the female body often with external reproductive structures, which are associated with fertility, e.g., the Venus of Willendorf found by József Szombathy (1853–1943) in 1908. It is now certain that people living in the Upper Paleolithic (30,000–10,000 years ago) did not aim to represent “Venus,” the symbol of feminine beauty (Figure 3). To date, archaeologists can only guess the role of these artifacts featuring nude women with crudely depicted body parts associated with sexuality, referred to as “Venus figurines.” The statuettes always lack facial features and feet, which implies that they functioned as symbolic representations of fertility (Dixson and Dixson, 2011).

Paleolithic people probably thought that women alone had the power of creating life through childbirth (Murray, 1934). This assumption is also supported by descent-related terms used in communities that have survived from non-literate cultures. The Australian Aboriginal concept of “spirit child” beliefs have fascinated anthropologists since the late 19th century (Merlan, 1986). Attributing the creation of a new life to totemic-territorial aspects rather than human contribution presuppose some disjunction between sex and reproduction. For example, among the Papuo-Melanesians of New Guinea, the natives of the Trobriand Islands have no concept of biological fatherhood, which is reflected in their language. The word they use to refer to the father (*tama*) literally means the “husband of my mother” (Malinowski, 1932). Practically, this term tends to denote the man who raised the child in his loving and protecting company (Sider, 1967). Trobriand islanders believe that a woman conceives children when a matrilineal ancestor spirit, a *baloma*, enters her body, thus men have no role in conception at all (Barnes, 1973; Chopra, 2001). In this respect, female fertility is also associated with additional mysterious forces in other Polynesian communities. The natives of the Marquesas Islands in French Polynesia attributed supernatural power to these forces which can frighten away gods and drive evil spirits out of the human body. During an act of exorcism among these islanders, a naked woman would sit on the possessed person’s chest and use her pudenda to drive evil spirits out of the sufferer.

African cultures also associate fertility with the female body, assuming that ancestralhood and heredity apply a canonical understanding that new-born children are an extension of deceased ancestors (Gable, 1996). For the Bijago islanders of Guinea-Bissau every living being, human, animal, and plant has an *orebuko* (Gallois Duquette, 1983). This principle inherited from ancestors endures after death and only women have the opportunity to be in contact with these spirits.^3^ Thus, motherhood is a central element in Bijago culture, since woman come into contact with the spirit world every time a new life is created in the womb. The power over life has created a matrilineal society among Bijagos where motherhood is venerated (Henry, 1993).

On the contrary, Homer’s poems, the *Iliad* and the *Odyssey* (ca. 800–701 BCE), attribute characteristics of men as inherited from their fathers (Stubbe, 1972). In medieval Spain, the surgeon, Abu al-Qasim al-Zahrawi, also known as Abulcasis (936–1013), attributed hemophilia to heredity in his *Al-Tasrif* (Cosman and Jones, 2008). A few generations later, the Spanish Jewish scholar, Judah Ha-Levi (ca. 1,075–1141) observantly wrote:

We perceive a similar phenomenon in nature at large. Many people do not resemble their father but take after their grandfathers. There cannot, consequently, be any doubt that this nature and resemblance was hidden in the father, although it did not become visible outwardly (Ha-Levi, 1998).

While the ideas of maternal and paternal dominance directed thinking about generation for almost 1,500 years none of these ideas led to the development of solid theory of heredity.

## Pangenesis and the Power of Reproduction

The prehistoric focus on women had been replaced by the male-centered view—which persists in much folklore today—that sperm or “seed,” the only immediately apparent product of copulation, was responsible for fertilization. However, the appearance of particular quantitative traits, such as height, which often manifested in the offspring as a combination of the two parents, remained a mystery. For example, a tall man and a short woman had a child of average height. These types of observations gave rise to the theory of “blending inheritance.” To explain the phenomenon of heredity, two philosophical traditions developed in ancient Mediterranean Europe: one emphasizing particles, called pangenesis; the other emphasizing the material nature of heredity and the added role of the environment. Stubbe’s (1972) excellent book should be consulted by readers eager to learn the details. Herein, we summarize what we consider are the highlights.

Credited with being the Father of Medicine,^4^ Hippocrates of Kos (460–377 BCE), hypothesized that “*minute particles from every part of the body entered the seminal substance of both parents*, *and by their fusion gave rise to a new individual exhibiting the traits of both of them*” (Orel, 1996).^5^ In this respect, both parents produced “semen” or seminal fluids that intermingled to create the embryo. Mixed traits were explained by the blending of male and female seminal fluids, and the sex as well as characters of children to be born were thought to be determined by whether the paternal or maternal “seed” became “dominant” during the mixing that followed copulation. It was thought to be the reason why some children inherited the mother’s eye color, while others inherited the father’s hair color. On the other hand, the intermingling of equal proportions of male and female seminal fluids produced the traits of both parents in offspring. According to Hippocrates “*the seed comes from every part of the body, healthy seed from the healthy parts, diseased seed from the diseased parts*” (Orel, 1996). Stubbe (1972) suggested that this rendition of pangenesis was a modified version of older ideas that attributed the origin of semen to the brain or to the spinal cord. Probably influenced by Democritus of Abdera (460–370 BCE) pangenesis was popular through the medieval ages as an indivisible, or atomistic, view of the ultimate nature of reality. As for the location of the particles, the following basic ideas existed: the particles are carried by men, by women, or by both parents in the semen (other, non-materialistic ideas endured as well). The two-semen idea was also suggested by Epicurus (341–270 BCE), who proposed that the semen of males and of females were equally important in the formation of a new life.

**FIGURE 4:**
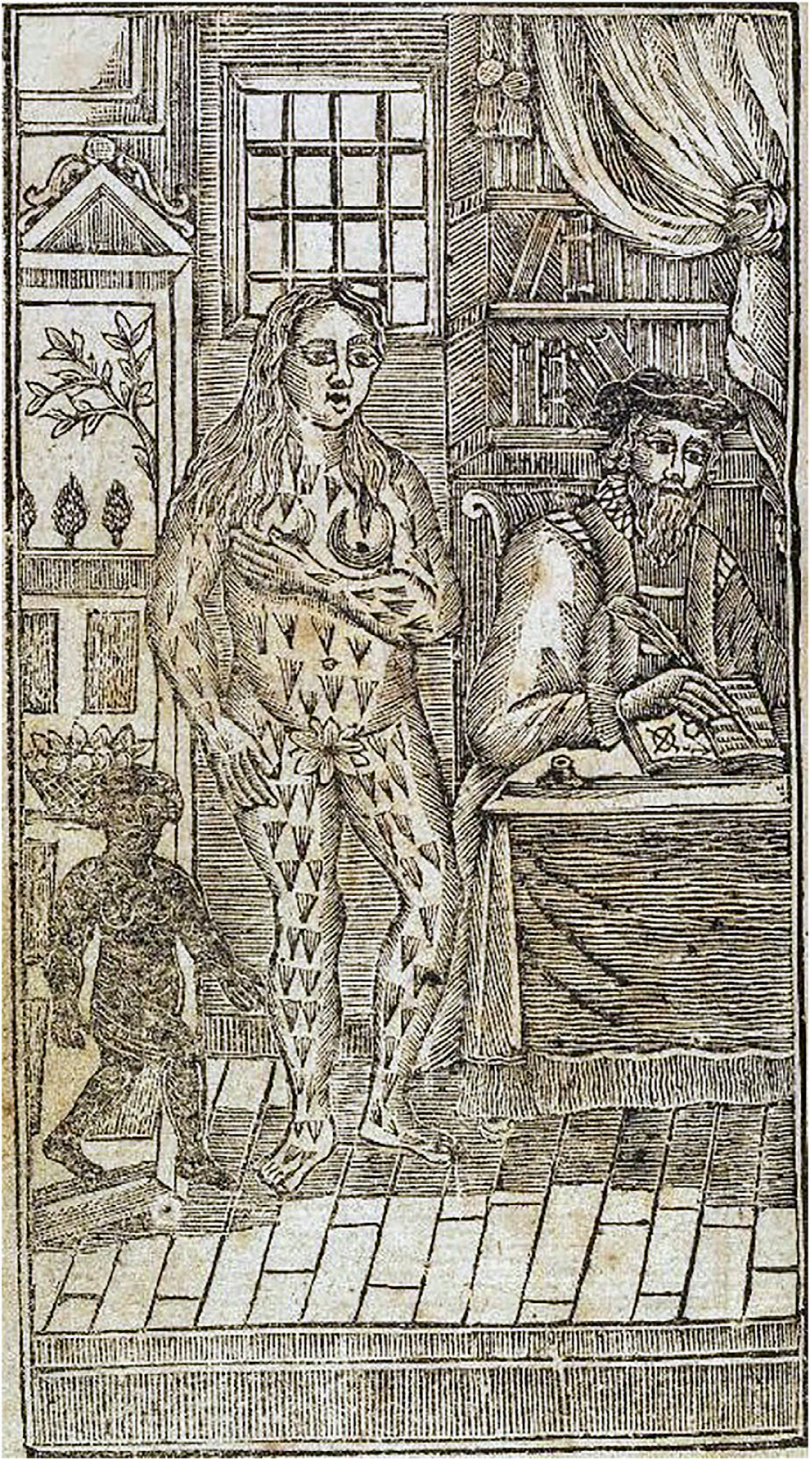
Aristotle unfolding the mysteries of nature in the generation of human beings. Printed for R. Ware, C. Hitch & J. Hodges (1749).

In contrast to pangenesis, Aristotle of Stagira (384–322 BCE), considered one of the founders of Western philosophy, proposed a more tangible theory (Figure 4). He thought that blood was somehow related to human reproduction. He thought that food is converted into a juice, or *ichór* (ἰχώρ) and a particle of our body is concentrated in a “nucleus” or germ (gemmule), which would be ultimately incorporated into the “blood.” Therefore, this nucleus, which of course is not to be confused with the eukaryotic cell’s nucleus, contained all parts of the body. Probably influenced by Pythagoras of Samos (ca. 570–495 BCE), Aristotle considered semen a purified form of blood converted from an excrement, or *perítto̱ma* (περίττωµα) and believed that the male’s seed “*acquired the active power to shape a new embryo*” (Leroi, 2010). For Aristotle, this meant that the germs in female menstrual blood were formed into a new living being by the movement of the paternal seed. Thus, the father’s seed induced qualitative changes in the mother’s reproductive matter. The existence of this “hematogenetic” theory, however, can already be recognized in the work of Parmenides of Elea, a philosopher roughly contemporary with Alcmaeon of Croton (Lesky, 1951). Aëtius, the writer of a description of the opinions (*placita*) of philosophers (what we now call a doxography), also attributed a “hematogenetic” theory to (perhaps still earlier) philosophers of the Pythagorean circle (Lesky, 1952; König, 2019). Aristotle also understood that hereditary matter is not reproduced but particular unchanged characters are transmitted across generations. True to his hylomorphic view of reality, along with matter, the visible form, or *eidos* (εἶδος), of a species was carried by the male’s semen (Quarantotto, 2010). Different environmental attributes, such as temperature, age, diet, etc., affected the gender of the newborn. For instance, Aristotle and others before him also thought that viscous and/or warm semen was conducive to produce male babies (Stubbe, 1972).

This ideology constituted the basis of speculation in the ancient study of heredity. The incorporation of a role for the environment in genetics resembles the more modern idea that the genes and the environment contribute to the phenotype directly (Lehoux, 2017) or through epigenetics (not to be confused with epigenesis). Also, to Aristotle and many others, the environment alone could also produce some forms of life. Centuries later Virgil’s *Georgics* called attention to this ancient fantasy by telling the story how mares were impregnated by the wind (see Zirkle, 1936). Aristotle and Hippocrates tried to unravel the process of generating life and explain similarities or differences between parents and offspring. Aristotle also understood that, as opposed to male-centered views of inheritance, maternal seed had a decisive effect on the offspring, just as plant seeds sown in different soils would produce plants with different forms, as he described in *Generation of Animals*. In the same work Aristotle also argued that nature has finite resources, thus it “*avoids what is infinite, because the infinite lacks completion,*” therefore, every deviation must be a degeneration of nature’s perfect form understood as a monstrosity (Peck, 1963). It is well known that such monsters frequently appear in sheep and goats. Aristotle refers to these monsters in the following terms:

Monstrosities occur more frequently in *goats* and *sheep*, because they are more prolific [whereby] the production of monstrosities has been already prepared for Nature by the fact that they generate offspring which, owing to its imperfect state, is unlike its parents:—monstrosities […] the *metáchoira* (μετὰχοιρα) are creatures which have in some respect undergone some “monstrous” affection […] (and they) belong to the class of “things contrary to Nature” (Lonie, 1981).^6^

However, neither Aristotle nor Hippocrates articulated deeper relationships regarding the hereditary effect of these monstrosities since they could not find any continuity between the properties of parents and their successive offspring. Although, Aristotle knew about the complexity of the environment surrounding all lifeforms, there is a valid justification found in his writings to speculate that he did not think that nature itself is capable of undergoing change. In fact, in his cosmological framework, the motions of the heavenly bodies determine the nature of sublunary conditions (Henry, 2006). For Aristotle, these celestial movements were eternal and perpetual. Epicurus of Athens (341–270 BCE) went further and combined Aristotle’s views and suggested that *atomoi* (ἂτoμoι) or atoms are combining to form *onkoi* (όγκοι) or bodies and in case of living organisms both sexes contribute to heredity (Yapijakis, 2017). Asclepiades of Bithynia (124–40 BCE) following the Epicurean philosophy associated diseases with alteration in the atoms of the parent’s molecules, thus describing hereditary diseases.^7^ One of the followers of Asclepiades’. Soranus of Ephesus (first/second century CE) described further hereditary and inherent mental disorders.

The great–as sometimes he is called–Galen of Pergamon (ca. 130–200 or 210), whose views on the human body influenced European and Middle Easterners for centuries, attempted to synthesize and rethink the ideas of Hippocrates and Aristotle in a body of works that reflected the pragmatic Roman world view. He adopted pangenesis, the two-semen hypothesis, and the notion that the balance (or lack of it) of the “bodily humors” partially explained health or disease (Cosman and Jones, 2008). Based on the theory of essential body fluids or humors, he divided people into four groups of temperament, an idea, which may seem to be ludicrous today. However, he recognized that certain phenomena of life and the laws that govern them can only be examined through experiments on living beings. Galen attributed great significance to the role of domestic plants and animals in studying these phenomena. He also attempted to formulate a hypothesis of sexual differentiation and inherited similarity in *De semine* (On Semen):

To think that a male animal is born when the male sperm prevails, is sufficiently plausible, but it is at variance with the fact that females are often very similar to their fathers, while many males are like their mothers. Perhaps, then, it would be better to say that it is not simply through prevailing of the different sperms that male and female *come to be*, but in relation to the differentiated parts. But this again, as I have said is in conflict with the fact that it is not only in the genital parts that the difference lies, but in the whole body (Lonie, 1981).

Galen combines the theory of pangenesis with sexual biopotency where both male and female parents contribute semen to form progeny. In antiquity, the questions of how generation took place and the contribution of each sex to creating offspring were generally heavily debated. It was even more obscure why progeny did or did not resemble their parents or how all this is possible at all. Although Hippocrates had stated that all organs in the chicken embryo developed simultaneously, Aristotle recorded accurate observations convincingly describing the order of appearance of different organs.

Aristotle and contemporaries hypothesized that spontaneous generation or abiogenesis explained the rise to living organisms from non-living matter. They based their evidence on observations, such as maggots produced from meat left opened, or mushrooms formed on decaying wood, and frogs seemingly originating from mud. There is nothing faulty about these individual observations, besides drawing causal conclusions from the two events occurring together in space and time that lacked causation leading to incorrect hypotheses about the mechanisms of heredity. As to the mechanism that caused such changes, St. Augustine of Hippo (354–430) scrutinized the idea of heredity. His works tried to reconcile Aristotelian teachings with Christian theology and directives of the Old Testament which, according to them, formulated that the world *came to be* from nothing through divine creation (*creatio ex nihilo*). He thought that “*God had endowed matter with certain powers of self-development, leaving the operation of natural causes in the production of plants and animals*” (see Lehoux, 2017). This theological view of nature slowly diminished its grip on scientists (Merz, 1965), but it took centuries for naturalists to argue that living things display characteristics quite distinct from those of non-living matter, making it necessary to understand processes rather than simply decompose phenomena to then analyze them (Castañeda, 1995).

## Heredity as a Stage in the Seamless Process of Development

**FIGURE 5:**
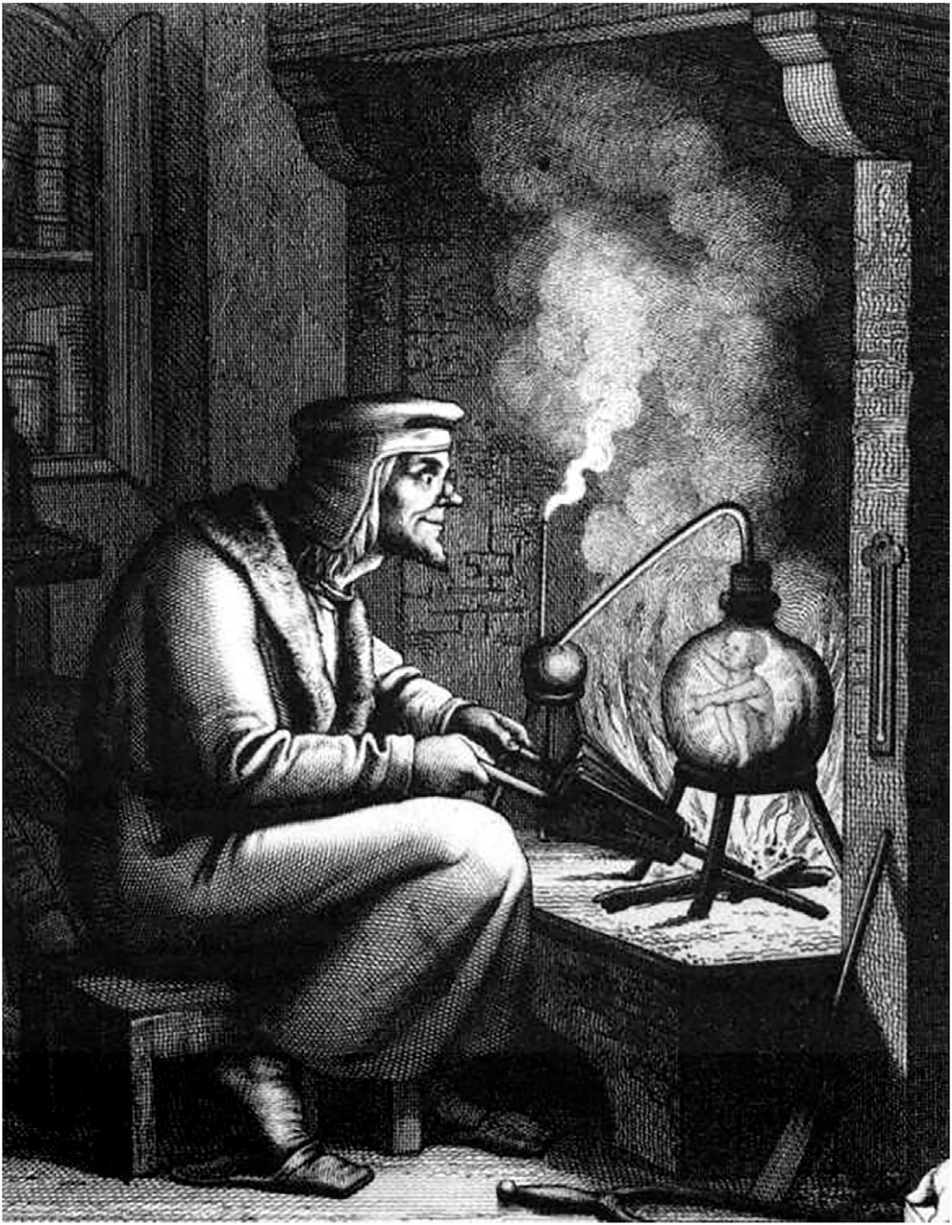
Alchemists also searched for the creative substance of human life. It was believed that the homunculus–the small human-like creature in the flask–could be produced chemically. This idea was later linked to heredity, and it was thought that there was a preformed homunculus in the germ cells. Source: 19th century engraving from Goethe’s Faust (Part 2). The alchemist and the homunculus.

In the 16th century, the French philosopher and scientist René Descartes (1596–1650) promulgated the use of reason and science to decipher the inner works of a machine-like Universe. The Renaissance slowly brought fundamental changes in scientists’ attitudes toward the Universe. Scientists realized that nature can be explored and explained if we understand the system of constitutive particles. All living beings were seen through mechanical laws. Isaac Newton (1643–1727) proved the existence of gravitational force mathematically, which created a need for studying the structures of the Universe, macrocosm and, later the natural world. It took only a short time for scientists to focus their attention on the world of the living, and they began to scrutinize what the mysterious “generation” (or procreation) really was (Figure 5). Additionally, the improvement of magnifying devices, such as the microscope [e.g., Robert Hooke (1635–1703); and Anton van Leeuwenhoek (1632–1723)], the gradual professionalization of scientists, the development of universities, and the increased communications between scholars catalyzed an intellectual upheaval in the sciences. Although the observations discussed herein came from the study of development and reproduction, they are also closely related to inheritance, since it is important to understand that the word “heredity” had no biological meaning in the 17th and early 18th centuries. The reader is referred to Needham (1959), Horder et al. (1983) and Farley’s classical work of Gamates and spores (1982) for fine-grained expositions of the history of embryology. Herein, we highlight only some key developments.

According to Stubbe (1972) and Needham (1959), the English physician, William Harvey (1578–1657) linked the origin of bird eggs to the concept of the mysteries of generation. He assumed that the mixture of unformed substances from both parents produced new progeny, and that fetuses were formed through gradual development, and lacking the characters of adults at an early stage. Besides, Harvey was the first to describe the system of blood circulation accurately, and his interest in embryology was not limited to experiments in eggs; he continued research on deer embryos. Harvey suggested that there must be some kind of “mammalian egg” too, and that all living creatures come from an egg. In search for this mysterious mammalian egg, he dissected dozens of does and hinds kept at the royal parks at Hampton Court. His findings were summarized in the book *Exercitationes de generatione animalium* (On the Generation of Animals), published in 1651. In this volume, Harvey described that deer embryos looked like little balls, which made him believe that he discovered some kind of “mammalian egg” during his dissection experiments. At about the same time, Harvey’s contemporary, the Italian anatomist Marcello Malpighi (1628–1694), studied the development of bird embryos and concluded that these embryos contained organs from the very beginning prior to the *punctum sanguineum* (point of blood).

**FIGURE 6:**
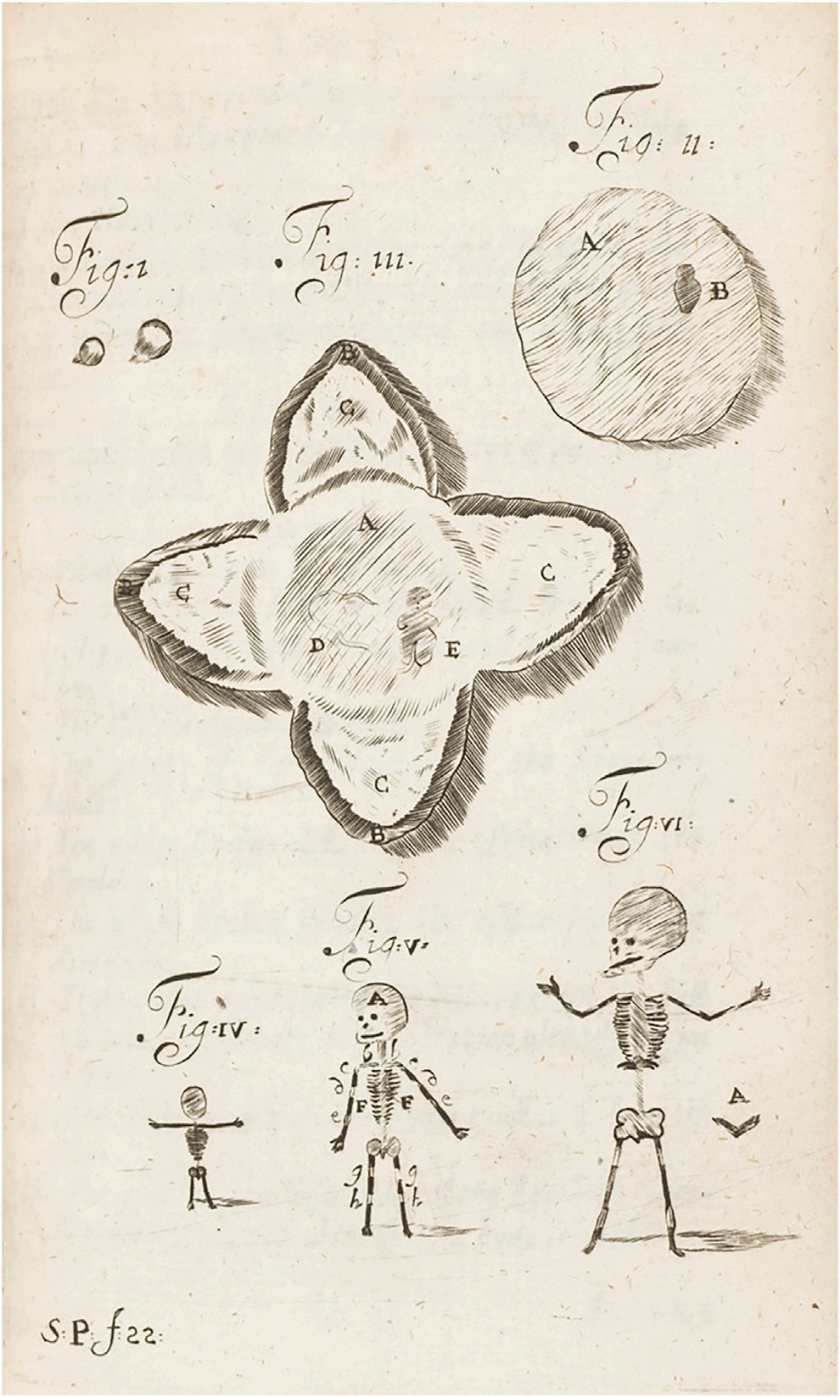
The preformationist “origin of man”. Woodcut from *The paradoxal discourses of Franciscus Mercurius Van Helmont concerning the macrocosm and microcosm, or, the greater and lesser world and their union set down in writing by J.B. and now published* (1,685). It vividly shows that preformationist thought that few weeks old embryos are fully formed children. In detail, figure I displays “two humane eggs of different bigness”, figure II “an Embryo of three, or at the most 4 days after Conception”, and figure III “placenta with the Veins and Arteries dispersed through the substance of it”. Figure IV “represents to the eye a gristly skeleton of an embryo of 3 weeks”, figure V “an Embryo of 1 month”, and figure VI, “an Embryo of 6 weeks” displaying a diminutive skeleton of a child.

For most embryologists of the time, the heart was the first organ that developed. Epigenesis (or development), represented by Harvey, on the one hand, and preformation (Figure 6) using Malpighi’s observation, on the other, gave rise to the first debate in embryology.^8^ Harvey stated that blood is made even before the heart was formed, an idea known as *punctum saliens* (leaping point), while Malpighi, based on many misinterpreted observations, insisted that the heart is formed before the blood and many other structures of the body develop from it. As Harvey wrote:

In a word I say,—from the *cicatricula* ^9^ […] proceeds the entire process of generation; from the heart the whole chick, and from the umbilical vessels the whole of the membranes […] that surround severally subordinate, and that life is first derived from the heart (Harvey, 1965).

Harvey’s epigenesis, a process that “*attracts, prepares, elaborates the matter and at the same time formation and growth appears,*” contradicted equivocal generation, the dominant view on the generation of living beings. For instance, in Europe, it was generally thought that barnacle geese, *Branta leucopsis*, grew on trees and that insects arise spontaneously from dirt. In fact, people also believed that, although rarely, women could give birth to animals or even monsters. For example, in the first half of the 17th century, the famous Flemish physician Jan Baptist van Helmont (1579–1644) advocating the idea of spontaneous generation published a recipe for generating mice *via* putting a dirty shirt, together with a handful of wheat into a jar. Van Helmont claimed:

[…] if a soul shirt be pressed together within the mouth of a Vessel, wherein Wheat is, within a few days (to wit, 21) a *ferment* being drawn from the shirt, and changed by the odour of the grain, the Wheat it self being incrusted in its own skin, transchangeth into Mice: and it is therefore the more to be wondered at, because such kinde of insects being distinguished by the Signatures of the Sexes, do generate with those which were born of the seed of Parents: That from hence also, the likeness of quality of both the seeds, and the like vitall strength of the ferments may plainly appear: And which is more. wonderfull, out of the Bread-corn, and the shirt, do leap forth, not indeed little, or sucking or very small, or abortive Mice: but those that are wholly or fully formed (van Helmont, 1662).

In the 1660s, the Royal Society of London, then seen as the world’s leading scientific body, discussed in precise terms how to produce vipers from wet soil (Birch, 1756). In a world view where mice could be generated from the mixture of a dirty shirt and a handful of wheat, it was difficult to argue rationally about the related issues of reproduction and heredity. The very use of the word “reproduction” in relation to generation became widespread only in the second half of the 18th century; thus, even the simple study of this topic seemed to be a stillborn idea in a milieu where insects could just spring from dirt. It was the unquestionable–and soon to be demonstrated, so very wrong–order of nature.

**FIGURE 7:**
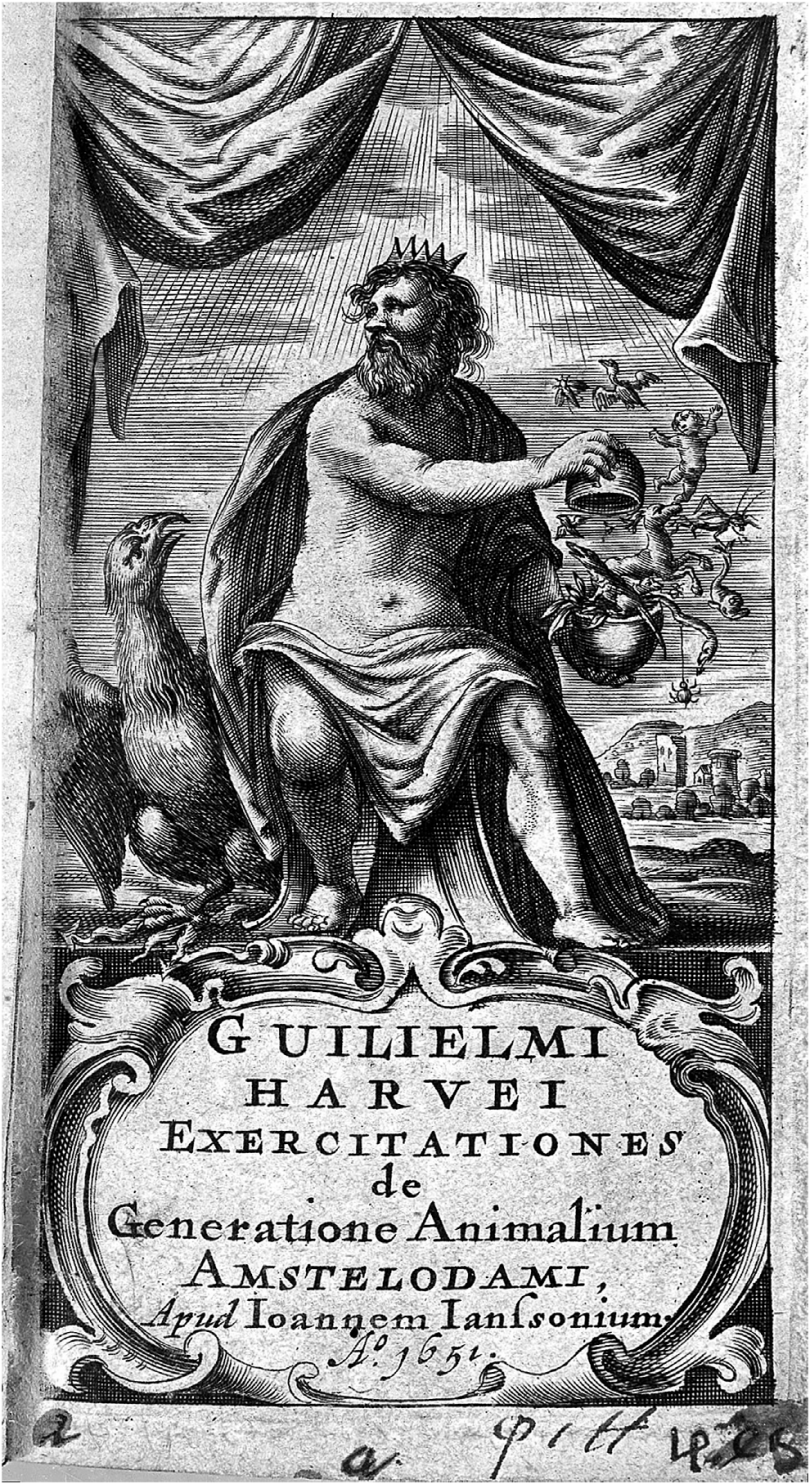
*Ex ovo omnia*. The frontispiece probably etched by Richard Gaywood for William Harvey, *Exercitationes de generatione animalium: Quibus accedunt quaedam de partu: de membranis ac humoribus uteri de conceptione* (1,651) depicts Zeus holding an egg in his hands from which all types of living beings emerge. Harvey insisted in his writings that all organisms emerged from the displayed egg. Source Welcome Collection (L0006635).

Significant advances in understanding generation came in the second half of the 17th century, through the work of Regnier de Graaf (1641–1673), Francesco Redi (1626–1697), Nicolaus Steno (1638–1686) and Jan Swammerdam (1637–1680). Using theory, experimentation and dissection, these 17th century researchers proved that female organisms produced eggs (*ova*) and that “like breeds like,” including insects. The dictum “*ex ovo omnia*”, meaning, all life comes from an egg (Figure 7), which stood in contrast to spontaneous generation (Lehoux, 2017), became the assumption until Karl Ernst Ritter von Baer (1792–1876) discovered the ovum in female dogs. While a Dutch draper and amateur naturalist Antonie van Leeuwenhoek discovered male gametes (*spermatozoa*) through a microscope, although the term spermatozoon was coined subsequently by von Baer. When Leeuwenhoek examined human and (mainly) rabbit sperm through his microscope, he concluded that, with proper magnification, little spermatic animals, or “animalcules” (*Samentierchen*), became visible. However, Leeuwenhoek thought that eggs served only to feed and develop the resulting preformed fetuses.

In the seventeenth- and/or 18th centuries, scientists disagreed on what gamete was mainly responsible for the process of *coming to be*. Two schools of thought had developed in Europe proposing to explain the changes in organisms through time or development (Horder et al., 1983). The discovery of sperm and ovules—a riveting story by itself—led some scholars to believe that organisms were contained within one of the gametes, in miniature.^10^ Therefore, in humans and in other organisms as well, development consisted of the unpacking and growth of the homunculi, which was regarded as a mere mechanical process of unfolding and growth into the final form of the organism. This idea is known as preformation or preexistence. Since the forms were already organized, and the unfolding was referred to be undertaken by God himself. At the act of creation, order had to be imposed by God, thus the process was out of scope for further scientific investigations.

**FIGURE 8:**
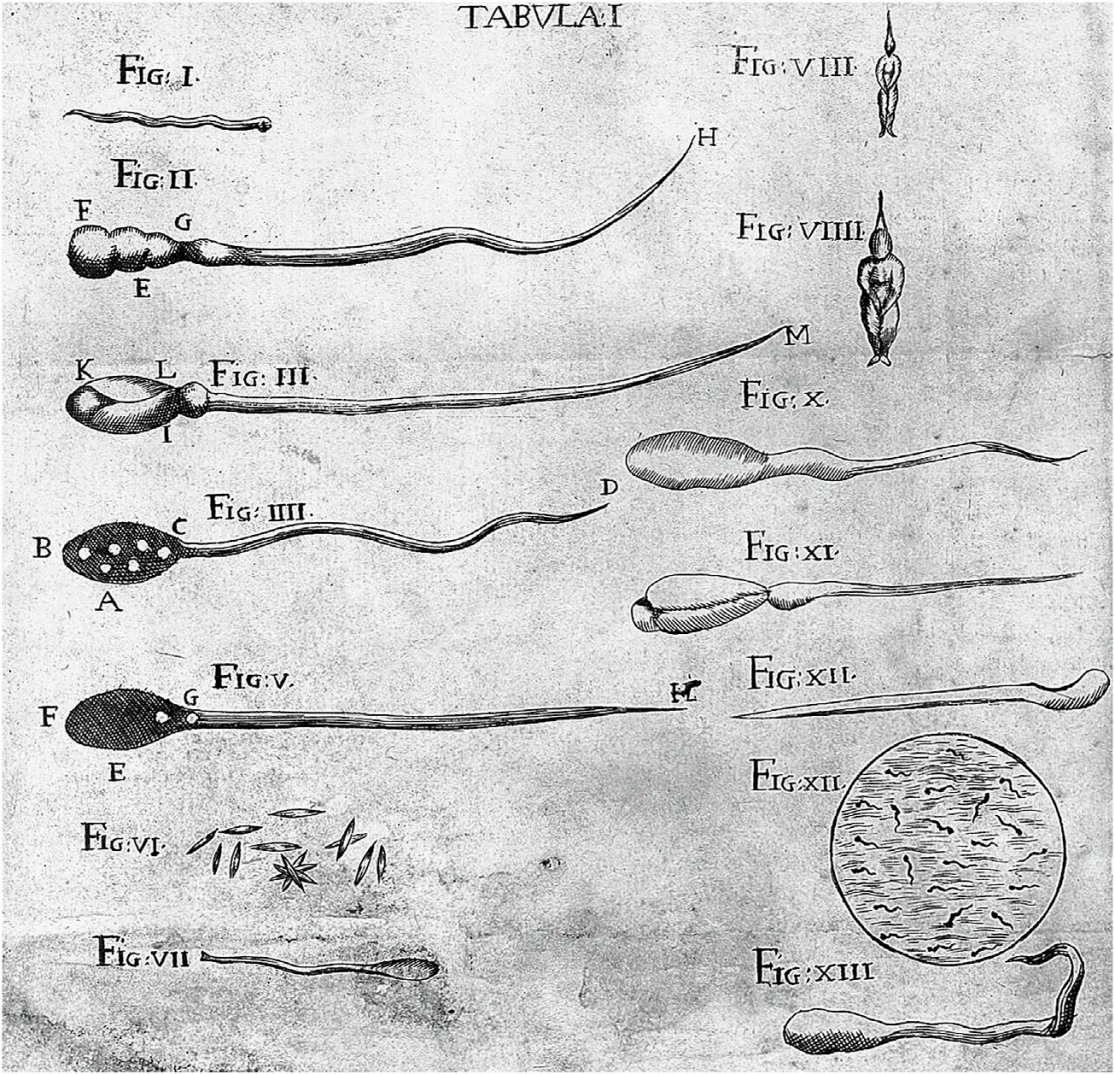
Illustration of animal sperms. Drawings from Antonio Vallisnieri’s book entitled “*Istoria della generazione dell’uomo, e degli animali, se sia da’vermicelli spermatici, o dalle uova* (1721)”. Note that Vallisnieri’s Fig. VIII and Fig. VIIII depict the preformed homunculi residing in the “head” of the sperms intersected in Fig. III and V.

As for the location of particles carrying the homunculi there were two camps: the spermists and the ovists. No one in the 17th century seems to have assumed that ovule and sperm were complementary elements within the same process and made equivalent contributions to the offspring, just like in intercourse. The scientists of this age had failed to reach this conclusion; instead, they had been debating, for almost 150 years, which component was the key factor, the egg (ovists) or the sperm (spermists). For instance, spermist scholars favored the notion that the sperm was responsible for the new being, the ovule served as food. In contrast, ovist scholars favored the notion that the ovule was the essential component (Figure 8). Some ovists, such as von Baer, thought that spermatozoa were parasites. Later, in 1745, this controversy produced the concept of “reproduction” and novel explanations emerged as an alternative to preformation (Roger, 1997).

## *Theoria generationis*: The Mystery of Generation

While the theory of preformation assigned the organization of particles to God *ab Origine Mundi*, other alternative explanations implicated that this was done through specific organic forces explained by versions of epigenesis. These concepts provided an explanation that elements of the organic body interact and, rather than going through a mechanical unfolding process, the heterogenous mixture is formed by non-mechanical forces associated with matter. Recent developments in the study of gravitation and chemistry made it possible for proponents of epigenesis to associate such forces with organic matter (see Terral, 1996). However, the explanation of inheritance seemed impossible for naturalists. Moreover, when the microscopic world opened up, they discovered still more unintelligible examples and inexplicable phenomena, which induced uncertainty and controversies. One of these phenomena was the incredible ability of some animals to regenerate–literally repeating the process of generation. From the 1680s naturalists were stunned by the fact that lizards can regenerate their lost tails, as first described by Melchisédech Thévenot (1620–1692) and Claude Perrault (1613–1688) both members of the French Academy of Science. These observations created problems in the “science of generation.” The question arose as to how could such a precise structure as the tail *come to be* from undefined matter without any guiding force? Perrault’s answer relied on the *emboîtement* theory, popularized by Jan Swammerdam (1637–1680) that preformed “germs” are present in all parts of the body (see Bowler, 1971). Thus, Perrault accepted the Leibnizian notion that all parts of the organic machine possess vital properties (Benson, 1991). Later in 1712, René-Antoine Ferchault de Réaumur (1683–1757) postulated that these invisible “germs” are scattered through the body as part of the original creation and, thus, their quantity is limited, meaning that body parts cannot regenerate indefinitely. He based his theory on the regeneration of amputated crayfish claws (Réamur, 1712).

**FIGURE 9:**
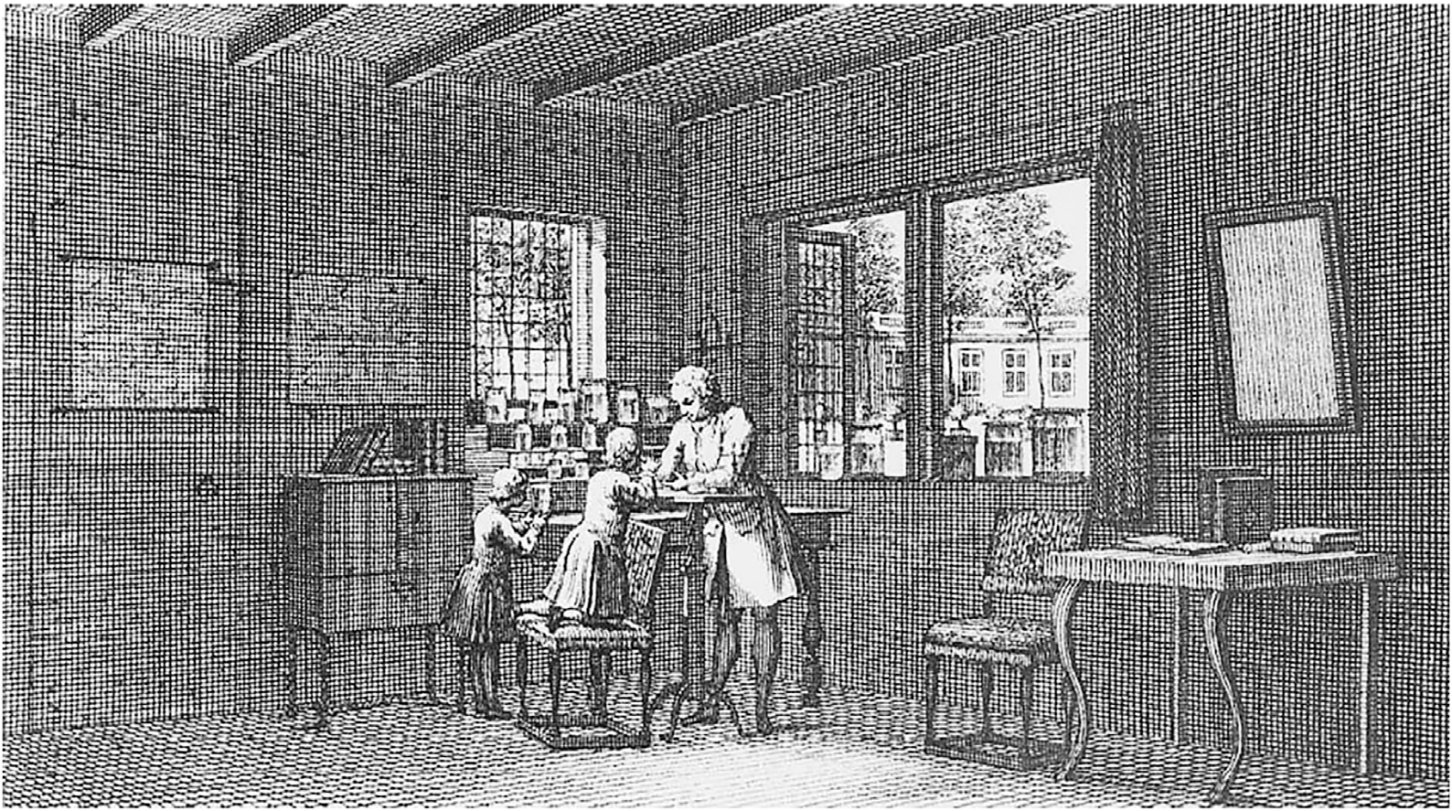
The laboratory of Abraham Trembley. The depiction appears in his *Mémoires, pour servir à l’histoire d’un genre de polypes d’eau douce, à bras en forme de cornes. Leiden: Chez Jean and Herman Verbeek, 1744*.

**FIGURE 10:**
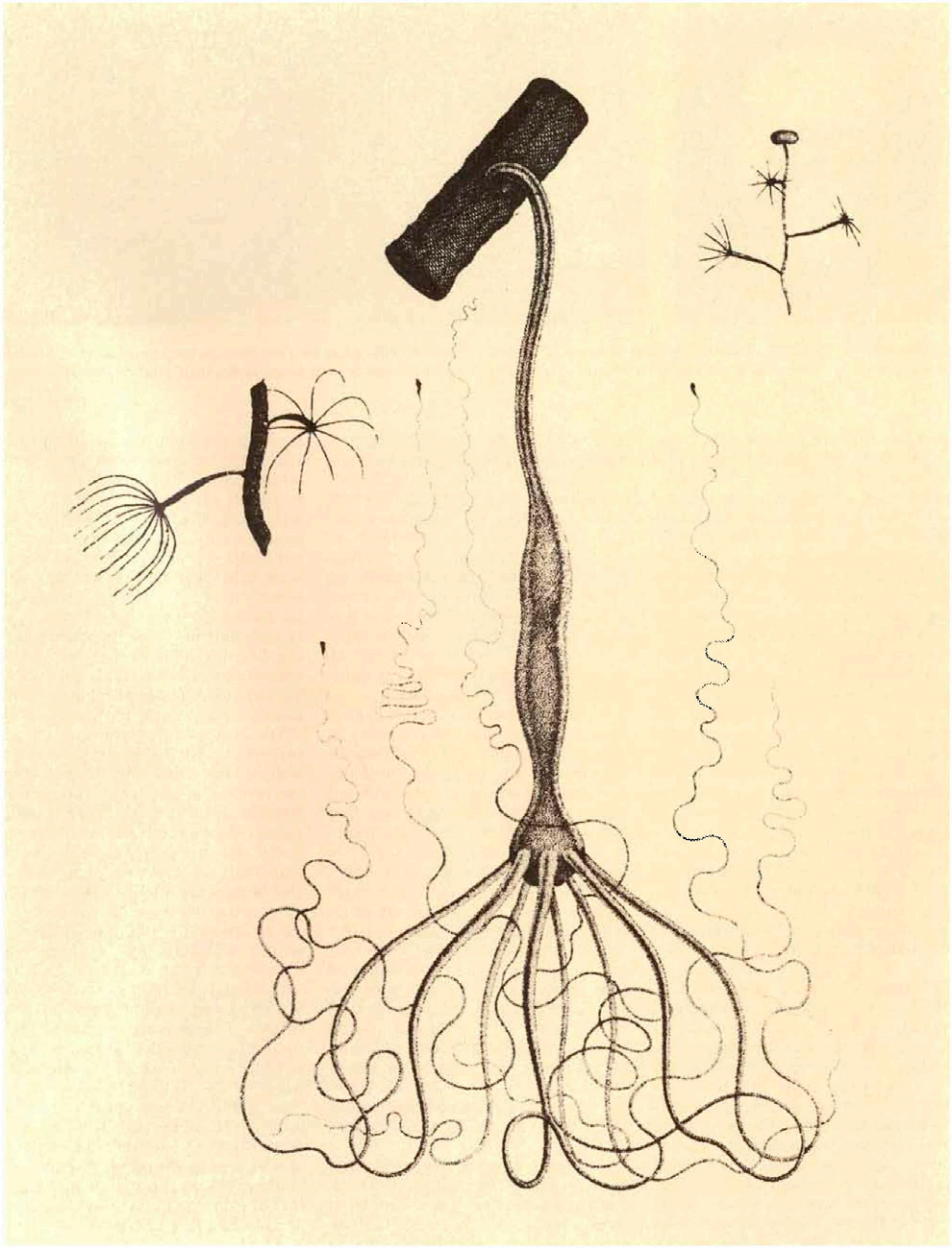
The drawings of the naturalist by Pierre Lyonnet (1706–1789) of the *Hydra* studied by Abraham Trembley. The drawings were published in his Mémoires (1744). The animals were named in 1746 by Carl von Linnaeus (1707–1778). Trembley originally studied the green species on the right, *Hydra viridissima*. In his later work, he also studied two other brownish species, *H. oligactis* in the middle and *H. vulgaris* on the left.

The discovery of the *Hydra*, a cnidarian with a simple body plan, by Abraham Trembley in 1741 inspired further speculations about the organization of matter and theory of generation (Figure 9). Trembley first thought that the discovered creature was a plant and proceeded to cut it in half, assuming that the two parts would continue living similarly to other plants. To his surprise, the *Hydra* regenerated its entire body from the two halves, which, to say the least, was the most unexpected phenomenon seen so far by a naturalist.^11^ The seemingly miraculous regenerative abilities of *Hydra* inspired many questions among scientists: If *Hydra* has two heads does its soul multiply with it? Is *Hydra* soulless or are new souls and creatures created during the regeneration? Are there any other analogies to be found in nature and how does this relate to the theory of generation? Is *Hydra* an intermediate primitive creature, a link between plants and animals? Trembley was not interested in investigating such questions, instead he further observed *Hydra* and described its feeding habits besides its natural reproduction through budding (Trembley, 1744; Ratcliff, 2004). His report, however, inspired many other naturalists to search for similar phenomena in nature. *Hydra* was instrumental in converting some scientists to support epigenesis and it became a central motif of materialist theories on epigenesis. In the same year of 1741, the Genevan naturalist, Charles Bonnet (1720–1793) discovered that freshwater worms can regenerate two identical organisms like *Hydra* when they are cut into halves (Figure 10). Bonnet published his observation in 1745 in his book entitled *Traité d’insectologie* together with the observation of parthenogenesis in aphids (Lang and Santiago-Blay, 2012).^12^

Unfortunately, Bonnet’s discovery of parthenogenesis among aphids undermined Réaumur in his views on parents and traits transmitted through their “particles”; he subsequently abandoned his attempts to observe the phenomenon of polydactyly among humans.^13^ Before the aphids, he assumed that reproductive matter contained the parents’ organic molecules where, after a union driven by special forces, a new order emerges, thus the offspring is formed. The transmission of traits is linked to this process. He remained clueless; how can anything be inherited if aphids can reproduce without mating? These ideas were further elaborated by mathematician and astronomer Pierre-Louis Moreau de Maupertuis (1698–1759) who was also inspired by *Hydra* to speculate about the question of generation. In his anonymously published book *Vénus physique* (1745) he wrote:

What are we to think of this strange kind of generation? Of this principle of life spread throughout each part of the animal? Are these animals anything other than collections of embryos just ready to develop, as soon as they are allowed? Or do they reproduce by unknown means all that the mutilated parts are missing? Might Nature, which in all other animals attached pleasure to the act by which they multiply, have caused these [creatures] to feel some kind of sensual delight when they are cut into pieces (Maupertuis, 1745)?

Maupertuis also went on to study polydactyly, like Réamur, but in albino children. His observations led him to conclude that both parents contributed “particles” to the offspring in equal proportions, and that their disproportionate contributions would bring about “monsters.” Maupertuis suggested that in albinism, as well as polydactyly, the trait of interest could be caused by a change in the parental particles during transmission. This alteration was conspicuous, since all the parents of albino children were black. Maupertuis’s theories were rejected by his contemporaries because he adopted views that derived mainly from Greek atomist notions and he dismissed both the ovist and spermist ideas mentioned above (see *Heredity as a Stage in the Seamless Process of Development*). However, this could not discourage either Maupertuis or Réaumur from proceeding with their studies of polydactyly among the descendants of German and Maltese families (Glass, 1947; 1955). Looking at the way this character reappeared over the generations, both scholars arrived at conclusions that were sharply opposed to preformationist theories. Maupertuis even calculated the probability (8×10^12^ to 1) that this trait would not be transmitted over the three consecutive generations (Sandler, 1983). Up until the late 19th century, the application of mathematics to the phenomena of biological heredity could be considered a remarkable novelty. It is apparent that Maupertuis discussed the phenomena of dominance and segregation of particles and used probability to investigate the transmission of an inherited trait among generations.

**FIGURE 11:**
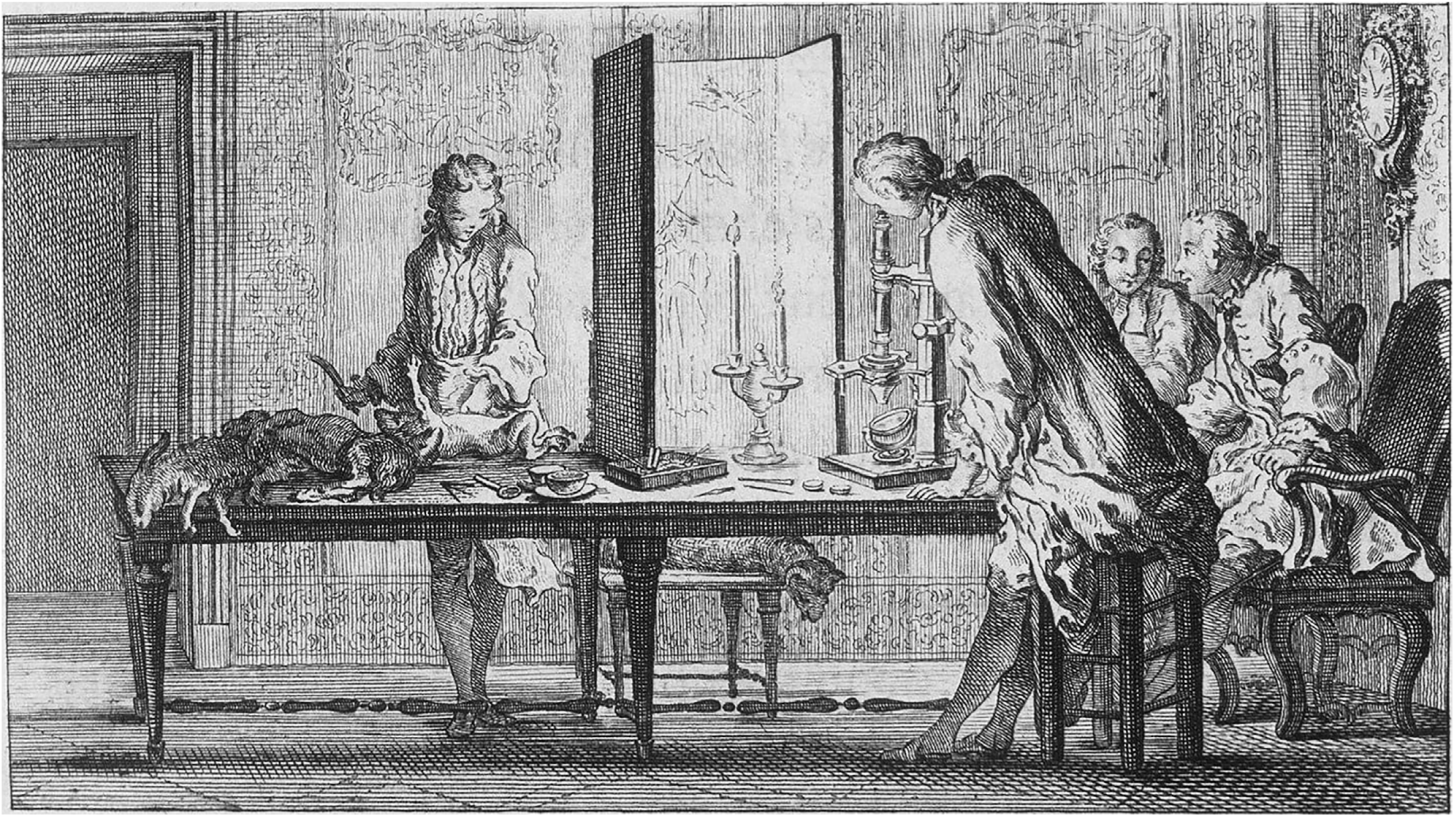
Search for homunculi in the female seminal fluids. The figure shows, from left to right: an unknown figure, Louis-Jean-Marie Daubenton (1716–1800), Needham, and Buffon. Source: *Histoire naturelle*, vol. 2. (1749).

In sharp contrast to preformationists, Réaumur’s and Maupertuis’s work on polydactyly helped establish the principles of epigenesis, namely that development proceeds gradually from an undifferentiated structure, the zygote, not just through the enlargement of a preformed entity. *Hydra* also moved the imagination of Georges-Louis Leclerc de Buffon (1707–1788), who in a sense agreed with Réamur, but believed that particles were shaped into living beings by an internal order, the *moule intérieur* (Buffon, 1749–89). He also proposed that the formation of the embryo takes place after the seminal fluids are mixed: “*The embryo forms immediately upon the mixing of the fluids, then proceeding to grow by the assimilation of the organic particles through nutrition*” (Buffon, 1749–89). By rejecting the existence of germs Buffon, with the help of his colleague John Turberville Needham (1713–1781), tried to provide experimental proof that semen also exists in the female (Figure 11). This was based on the idea that organic particles are taken up by food and filtered by all parts of the body in the seminal fluids. Organisms collect such representatives of the body in their reservoirs, which are released during sexual reproduction. Further guidance is then given to these particles by forces similar to gravity, magnetic attraction, or chemical affinity. As Buffon wrote:

It is apparent […] that forces exist in Nature, such as gravity, which relate to the exterior qualities of bodies, but which act on the most intimate parts and penetrate to all points […] for, in the same manner as the force of gravity penetrates the interior of all matter, the force that pushes or attracts the organic parts of nourishment penetrates into the interior of the organized body (Buffon, 1749–89).

**FIGURE 12:**
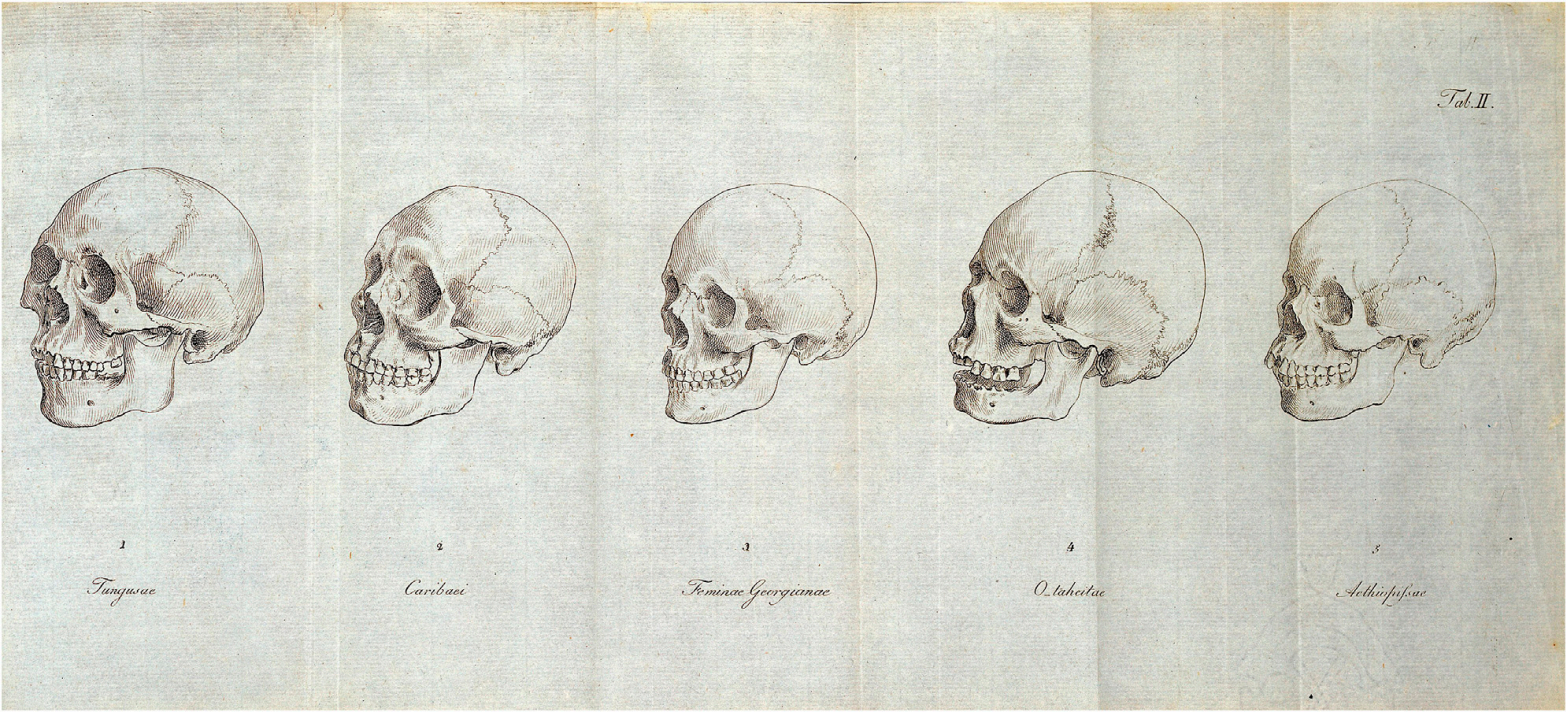
Blumenbach’s drawings of *De generis humani varietate* (1795). Table II depict false ideologies and beliefs on human races by showing a sequence of human skulls from so called “main types”. Like other monogenists, such as Buffon or Blumenbach, Wolff also assumed that the inherited entity appears in a variety of forms, which are primarily influenced by the environment and nutrition. To prove his theory, he measured and compared the skulls of thousands of people from different continents and countries in the 1750s. Based on his measurements, he divided people into races, suspecting that food, environment, and temperature are what determine the physical differences and transitions between different races. According to his theory, the Caucasian race, which is considered the natural starting point for Adam, could degenerate under the influence of inadequate food. As an example, he brought up the dark skin color of Ethiopians, which was created by the environment and removed from their natural environment in less sunny countries as the generations alternate, the hypothetical Caucasian basic race may re-emerge in their offspring.

This mystical order was later translated by Erasmus Darwin (1731–1802) into English as “penetrating power (force),” which German scientists began to refer to as *Kraft* directly translating the English word omitting Buffon (Gigantes, 2000). Subsequently, work by Rudolf Jacobus Camerarius (1665–1721) and by the German physiologist Caspar Friedrich Wolff (1733–1794) helped to establish the principles of epigenesis. As an early advocate and promoter of the idea of epigenetics, Wolff believed that the developing embryo carried a hidden entity transmitted from parent to offspring in the process of generation that he called “developmental history (*Entwicklungsgeschichte*).” He also supposed that this transmitted entity expressed itself in a variable form, depending on the influence of climate and nutrition. New physical variants *came into being* because of environmental influences acting on the seed of the parents guided by a specific force called the *vis essentialis* (Wolff, 1759). Wolff went on to carry out further work, which refuted the preformation theory, and tried to prove the existence of epigenesis, even trying to offer a confused explanation of variation and heredity (see Rajkov, 1964; Roe, 1979).^14^ He assumed that transferred matter was plastic, modified by the environment, and easily influenced by nutrition. In order to prove his hypothesis, Wolff took the case of Ethiopians and Europeans, asserting that “*with climate it is heat and its digestive force that have a strong effect on the formation of variations*” (Gaissinovitch, 1990) and “*[c]onstancies appear in structures … only because environmental conditions are common or remain the same*” (Roe, 1981). He argued that the digestion of different nutritive substances was dependent on environmental temperature, which determined the skin color in successive generations (Figure 12).^15^

## *Naturphilosophen* and the Genetic Force

While Wolff did not name his *vis essentialis* as a reproductive force, his successor, Johann Friedrich Blumenbach (1752–1840) of Göttingen, made the formative drive (force) (*nisus formativus* or *Bildungstrieb*) responsible for shaping biological reproduction. According to Blumenbach (1781), this organic force directed the formation and the appearance of an organism during reproduction and development in a physical and cultural sense. For scholars of the 18th century, heredity involved not only the transfer of traits from parents to offspring, but also a socio-cultural relationship (see Etzmüller and Tewes, 2016). Thus, Blumenbach’s formative drive was seen as an organizing force that explained the transformation of unorganized matter to an organized form in generation and human culture (Lenoir, 1980). Blumenbach believed that the differences between organisms exist due to their relatively distinct inherent features in their *Bildungstrieb*, which also prevented, for example, a dog embryo from becoming a cat during development (Richards, 2000). Blumenbach worked diligently on the further development of his theory (Blumenbach, 1782; Blumenbach, 1787; Blumenbach, 1788; Blumenbach, 1791; Blumenbach, 1797) and came to the following conclusion:

I hope it will be superfluous to remind most readers that the word *Bildungstrieb*, like the words attraction, gravity, etc., should serve, no more and no less, to signify a power whose constant effect is recognized from experience and whose cause, like the causes of the aforementioned and the commonly recognized natural powers, is for us a *qualitas occulta*. What Ovid said pertains to all of these forces—*causa latet, vis est notissima* [the cause is hidden, the force is well recognized]. The service rendered by a study of these forces is only that one can more carefully determine their effects and bring those effects into general laws (Blumenbach, 1789).

Blumenbach’s forces, on the other hand, remained closely related to epigenesis. Blumenbach initially shared the view that the essential elements of a developing embryo are already predestined, for example, in eggs. Later, he believed that the *Bildungstrieb* gave an actual explanation for the changes and was sharply different from another similar *Kraft*. He based this on the comprehensive architectural nature of his theory, which directed, organized and shaped the operation of physiological processes in detail, shaping the end product of development in the various organs and species.^16^

Blumenbach’s ideas fitted perfectly into the dynamically evolving ideas of science and medicine and embodied a physiology-based functional identity that philosophers and theorists of social processes have called potential or investment (*Anlage*). Immanuel Kant (1724–1804), a German philosopher from Königsberg who promoted the idea of the Enlightenment (*Aufklärung*) and the principle of *Sapere aude* (Dare to know!) also relied on Blumenach’s *Bildungstrieb* in developing his philosophical theories.^17^ Kant defined the financial term *Anlage* in a hereditary context as the source of something that needs to be developed (Kant, 1775; Kant, 1785). This biological interpretation made it possible to think of dispositions of hereditary traits as future potential, e.g., modified by the environment, but linked to an individual from the very beginning in the organization of ancestors (Müller-Wille and Rheinberger, 2007; Lehleiter, 2014). Kant explained development on a more heuristic basis while Blumenbach thought of a force derived exclusively of natural origin. The *Bildungstrieb* and *Anlage* quickly became the central theme of German nature philosophy (*Naturphilosophie*). This branch of philosophy examined the unity of nature and the possibilities of its interconnection (Nassar, 2010). In this theory, nature appeared to be the sum of what is objective, and wisdom or intelligence appeared to be the complex of all the actions that make up self-consciousness. The two complementary components that made up this philosophy as a whole were natural philosophy and transcendental idealism. Scientific criticism in the 19th century took hardly any notice of the distinctions between Romantic, speculative, transcendental, scientific and aesthetic directions (von Engelhard, 1988).

In addition to the environment and physical determinism, the effect of the environment on living beings and its transmission through the process of generation also appeared in the philosophy of Johann Gottfried von Herder (1744–1803) owing to Blumenbach’s inspiration. The stable entity of nature that *came into being* through the act of creation was a central theme in the 18th century, but it was also an important concept for the philosophers of antiquity where discrepancies were regarded as monstrosities (see *Pangenesis and the Power of Reproduction* and Zirkle, 1935). This type of degeneration from the original form was associated with hereditary diseases in the 18th century, where divine laws are equally valid for every life form on Earth particularly for animals and for humans since they all *came into be* through benevolent creation. The German philosopher Friedrich August Carus (1770–1807) stressed that:

Nature […] strives for the ideal; and human beings appear more and more the highest type on Earth […] mental sicknesses when human beings fall back into the state of animals through natural drives or madness—among which also belong the speechless wild children, and human beings retarded because of hereditary weakness, the Albinos of East India or Africa are unnatural appearances (Carus, 1809).

This anthropocentric view placed humans at the center of the world and passing on of traits relied on divine order not allowing for degenerate monstrosities caused by hereditary weaknesses. The idea of the genetic force (*genetische Kraft*) was at odds with such ideologies free of external factors, such as the theological pre-adaptation of epigenesis, which followed the guideline that living beings and their environment were created to be identical by predestination. Herder thought that an intrinsic and organic force, not the external effect of climate (*Klima*), is the ultimate creator of all living beings: “*The genetic force is the mother of all forms on Earth, which climate only works with in a friendly or unfriendly manner*” (Herder, 1785). This inner force for Herder had a shape-producing capacity that manifested itself in the forms it created. Different animal species, for example, he said are “genetically separated” (Herder, 1785). With a few exceptions, Herder’s notion of determinism is typically tempered by the extremely vitalistic genetic force, which resists climatic changes (Nisbet, 1970). The genetic force denotes some internal characteristics of the organism, which if altered can produce inherited changes, while other more superficial changes are not passed on (Nisbet, 1970). In this regard, Herder distinguishes between habitual deformities that people inflict on their bodies, e.g., nose piercing, foot binding etc., which have no lasting impact no matter how long they are practiced, while hereditary malformations can be inherited (Herder, 1785). Such hereditary characteristics can change, although only slowly and across generations, despite the fact that they are not fixed in time.

Herder also argued that ideas are influenced by the organization of the human body, and that the genetic force has a substantial impact on thoughts. He believed that fantasy and imagination (*Einbildungskraft*) were linked with the entire structure of the body generating human mythology, which was built out of experiences from the surrounding natural world (Herder, 1785). According to Herder, pictures and concepts acquired by humans living in a certain climate can be passed on to offspring as predispositions. Later, Herder wrote that generation plays a more important role in shaping human beauty than the climate (Herder, 1767), then went on to argue that the minds of unborn children are more than a blank slate because certain psychological qualities can be passed on to next generations (Herder, 1781). Because Herder considered his theory to be equally applicable to all living things, he used the term “genetic,” which is derived from the Greek word *genetikós* (γενετικός), and in this case it means connection or origins.^18^ Herder’s genetic force appears to bear the “genetic” adjective, but it should not be interpreted in an anachronistic way, although it is commonly related to inherited features that define various types of creatures.

**FIGURE 13:**
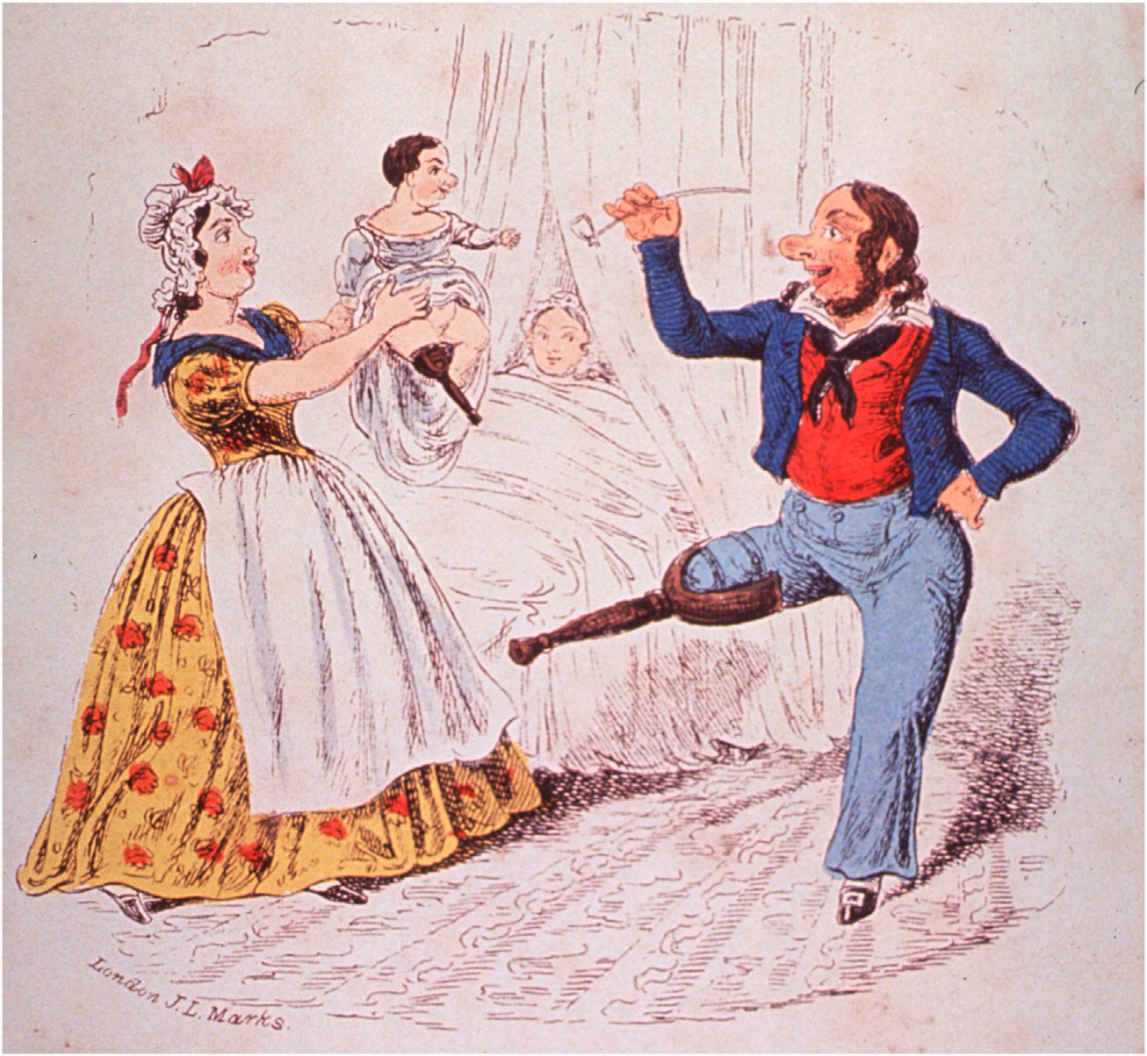
A chip off the old Block. A mid-wife holds up a new-born baby which has a wooden leg just like his happy father. The caricature of J. L. Marks (London, 1832) demonstrates well the contemporary thought about the inheritance of acquired traits. Image: National Library of Medicine, Bethesda, Maryland, United States.

The genetic force is the sum of the permanently inherited traits of living things before they are modified by external environmental factors. However, the biggest problem with Herder’s theory is that it somehow assumes the heredity of acquired traits in a Lamarckian way. It was Jean-Baptiste Lamarck (1744–1829) who claimed that giraffes stretched their necks because they had to gradually stretch higher to consume the foliage. Acquired traits were thus formed and further inherited in the descendants (see Lamarck, 1809; Zirkle, 1946) (Figure 13). Herder’s genetic force serves as the perfect basis for this direction. It is a philosophical concept not a biological theory. Despite the ambiguous titles used by Herder and other *Naturphilosophen* their monographs presented radical interpretations of nature that would echo throughout natural sciences of the next century, especially in biology.

## Medical Influences on the Study of Heredity

**FIGURE 14:**
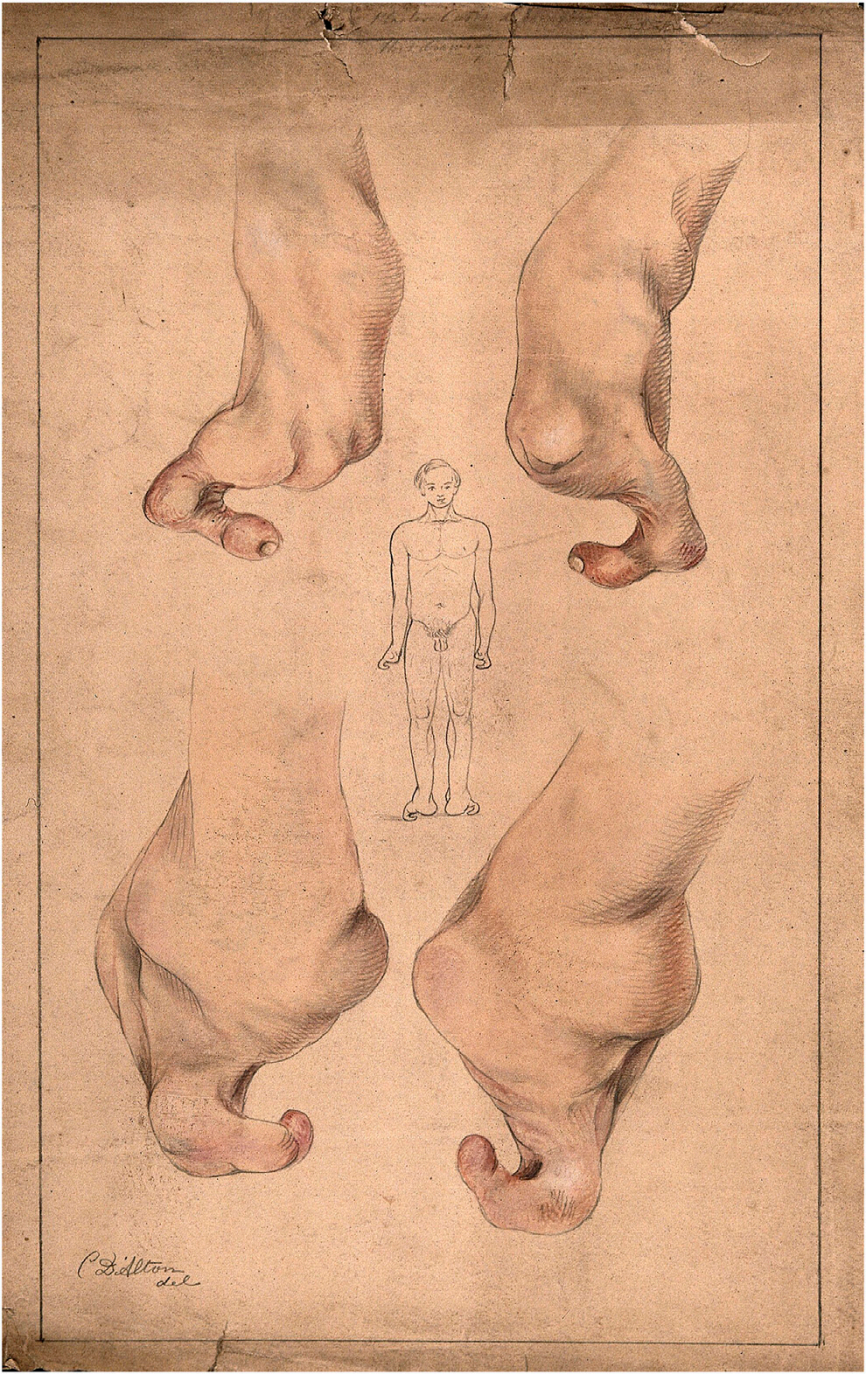
Passing on of “monstrosities” as physical deformations was well-known in the 19th century. The water colors and pencil drawings by Christopher D’Alton (1871) depict patients from the Royal Free Hospital, Grays Inn, London with congenital malformations.

The phenomenon of heredity did not go unnoticed by the end of the 18th century, as Müller-Wille and Rheinberger (2007) have shown, hereditary disorders (*haereditarii morbi*) and the passing on of physical monstrosities and behavioral peculiarities were attributed to some families (Figure 14). Medical doctors were documenting cases of insanity throughout Europe by the early 19th century, referring to heredity or “seed” as the most significant cause behind madness. This point stands out from the quote of the French physician Jean François Fernel (1497–1558):

Such as the temperament of the father is, such is the son’s, and look at what disease the father had when he beget him, his son will have after him […] as is as well inheritor of his infirmities as of his land (Fernel, 1554).

As Fernel’s famous quote illustrates, heredity was a metaphorical concept based on the contrast between the transmission of wealth as well as other legitimate goods and the general finding of physical resemblance between parents and offspring in this peculiar case, the undesirable similarity of illness. Heredity in human medicine was first expressed by the discovery that genealogical associations between people mean more than just social ties: there are also physical connections manifested by appearance and fitness (López-Beltrán, 1992).^19^ This was a novel concept that body and mind, or any of their characteristics, could be inherited in the same way as property, craft, and money. It took a long time for this concept to become ingrained in scientific biological thinking. Internal resemblances such as “temperament,” also reflected in Fernel’s quote but other behaviors or illnesses too, began being regarded as a valid source for hereditary investigations (De Renzi, 2007).

**FIGURE 15:**
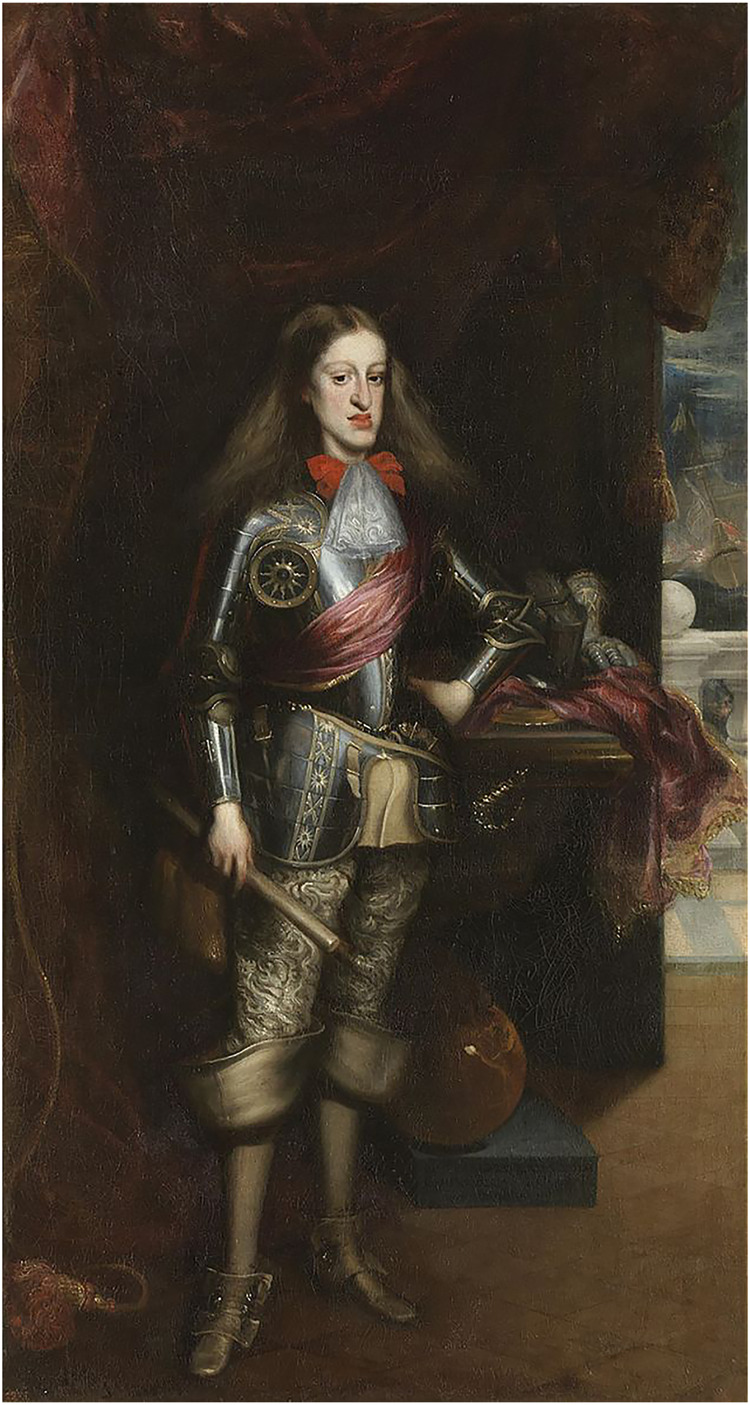
The son of Felipe IV (1605–1665) and Mariana of Austria (1634–1696), Charles II (1661–1700) was the last King of the Habsburg Dynasty in Spain. The portrait of Juan Carreño de Miranda (1614–1685) displays the peculiar heredity deficiency of mandibular prognathism and maxillary deficiency also known as the “Habsburg jaw” caused by inbreeding.

In the 18th and 19th centuries, debates by physicians regarding *haereditarii morbi* revolved around “temperament,” which corresponded to constitutional dispositions against particular diseases (Olby, 1993). Physicians were also concerned with linking insanity to family genealogy and preventing relationships from having malignant results (Porter, 2018). Since for the public, aristocratic families were synonymous with diseases, such as epilepsy, apoplexy, and mania; they also attempted to stop the spread of hereditary defects well known among their sphere. Gout, for example, was believed to be passed on with royal names, and “degenerative” ailments, like *tuberculosis* or madness were also associated with the upper crust (Olby, 1993; Wilson, 2007). Because of matrimonial policy to create political alliances by marriage, European royal dynasties of the Early Modern Era arranged consanguineous marriages, such as between uncle and niece, first cousins, and other such unions (Ceballos and Álvarez, 2013). The Habsburg family (also known as the House of Austria) was well known for hereditary propagation of illnesses or peculiar physical characteristics, such as mandibular prognathism. This facial feature, also known as the “Habsburg jaw,” was present in at least nine generations of the family, most likely as a result of inbreeding (Hodge, 1977; Vilas et al., 2019). Recent research showed that this peculiar physical feature inherited by many members of the family was caused by the co-occurrence of maxillary deficiency (MD) and mandibular prognathism (MP), which resulted in pronounced lower lips and overhanging nose tips (Álvarez and Ceballos, 2015). The pedigree-based inbreeding coefficient (F) revealed a statistically important association between MP and MD among Habsburgs, suggesting that inbreeding among family members triggered this disorder. Consanguineous family partnerships reduced general genetic fitness and increased the risk of recessive allele inheritance in the offspring (see Vilas et al., 2019). Contemporary genetic research has shown that Charles II (1665–1700), also known as “The Bewitched” (*El Hechizado*), was so disfigured, as a result of fatal inbreeding that the male line of the Spanish Habsburgs was wiped out (see Álvarez et al., 2009; Álvarez and Ceballos, 2015) (Figure 15).

In the 18th and early 19th centuries, doctors also discussed the inheritance of such features, as well as other “degenerative” diseases and advised noble families how to avoid them—with more or less success. Although, this need drew increasing interest from physicians resulting in highly esteemed prizes and competitions held by the Royal Society for Medicine (Paris) in the 1790s (López-Beltrán, 2007). These competitions included numerous debates about congenital, connate, and acquired diseases, observational parameters regarding the timing of the occurrence of hereditary diseases (*homochrony*) or the interpretation of delay (latency) when observed diseases skipped a generation (López-Beltrán, 2007; Müller-Wille and Rheinberger, 2007). The discussions outlined the biological definition of *hérédité* (Lópéz-Beltrán, 1994), which soon became widely applied by French doctors. The metaphorical adjectival use of heredity also became well established by the 18th century in several forms in multiple languages (Thomson, 1908; Zirkle, 1946; David, 1971). This adjective was transformed into a noun in English only between the 1860–70s to correspond with the French *hérédité* (Thomson, 1908; Lópéz-Beltrán, 1992). This was especially needed since by the 1870s, evidence was emerging around the new cross-cutting scientific domain associated with the scientific use of heredity.

The medical disclosure about the question of heredity—sparked by chronic health issues—soon developed into criticizing the aristocratic family form by the landless poor (Pomata, 2003). Hereditary aristocracy was founded on the concept of transmitting the “noble spirit” or “noble blood” from one generation to another, which necessitated the transmission of pure bloodlines free of interclass marriage (Wilson, 2007). Mixing of “noble lines” with “common blood” was believed to cause degeneration and the loss of noble features. Noble features were ridiculed by the rapidly increasing classes of the landless poor who considered themselves to be the subjects of this hereditary hierarchy (Carton, 2007). The upper class, for their part, saw these movements as a potential obstacle and a dangerous threat to the social order resting on hereditary titles (Pomata, 2003). Based on these examples, it is now part of the common historical narrative that the biological understanding of heredity arose in part from studies of human illnesses handed down from generation to generation. The numerous and conflicting examples arising from medical, philosophical, and from a natural historical perspective were ultimately less insightful than the evidences accumulating in practical agriculture. While ruling houses and nobles sought to avoid the negative consequences of heredity, people working in agriculture, specifically in animal breeding chose a different path and tried to “harness the power of heredity” to their advantage.

## *Theoria Cum Praxi*: The Genetic Laws of Nature Before Mendel

In the mid-1700s, Spanish Merino sheep were brought to England for the benefit of the newly established textile industry. Robert Bakewell (1725–1795), a Leicestershire farmer who pioneered systematic selective inbreeding of livestock took care to raise his sheep in similar circumstances and to breed only those who acquired the greatest weight while using the least feed (Wood, 1973). Among other things, Bakewell discovered that sheep cannot generate equal amounts of mutton and wool (Wood and Orel, 2001). For further details see our recent review Poczai and Santiago-Blay (2021).

Bakewell’s systematic approach led to a surge in demand for Merino breeds. Between 1768 and 1775, the early imports of Spanish sheep into the Habsburg monarchy resulted in the establishment of imperial stud farms that served as seed-stock dispersal hubs for improved flocks. The most significant impact on the advancement of Merino breeding came from a group of wealthy landowners and breeders in Central Europe (Brno Moravian, now part of Czechia and Kőszeg, Hungary). Ferdinand Geisslern (1751–1824) of Hoštice, a modest estate on fertile terrain northeast of Brno, was a prominent sheep breeder. In a flock of 400–500 animals, he used sophisticated methods, such as specialized recording, intensive selection, and inbreeding (Orel, 1997). This flock quickly became the well-known in the Habsburg Empire, and Geisslern acquired the name “Moravian Bakewell.” But Geisslern was not alone. Imre Festetics (1764–1847) also began experimenting with inbreeding in sheep and other animals on his estates near to Kőszeg (Hungary) from 1803 to 1847 (Szabó, 1991).

Christian Carl André (1763–1831) was a pivotal player in the development of natural sciences in Moravia during this period. André relocated from Saxony to Brno in 1798, where he established a reputation as an accomplished educator and author of natural science papers. In 1806, as secretary of the newly formed Moravian Agricultural Society (MAS), he suggested that the Academy of Science and the Society of Economics collaborate to advance both theoretical and practical research. André was named secretary of the “Sheep Breeders Society” (SBS) in 1814, a division of the MAS dedicated to developing scientific techniques for improving sheep breeding for the benefit of the textile industry (Orel and Czihak, 2001). He created a broad and fertile atmosphere of inquiry that encouraged discussion about the scientific foundation for breeding techniques, which included artificial selection methods and the transfer of wool characteristics from parents to offspring—in other words, heredity (Orel, 2009). From 1811, André published the weekly journal *Oekonomische Neuigkeiten und Verhandlungen* (ONV, Economical News and Announcements), which disseminated 6,000 copies across the Austrian Empire.

Between 1817 and 1821, André organized meetings in Brno to understand the theory controlling inbreeding. He referred to this as “physiological natural laws,” in which unqualified pairing in closest consanguinity leads in the organism’s deterioration over time. He also addressed fifty “urgent” concerns about breeding techniques in his notes (André, 1818a,b). Herein, we list a few of André’s questions: Is the idea of inbreeding already understood? What does weakening mean? Does weakening influence the constancy of wool fineness? Is weakening linked with disease susceptibility? Does weakening have an effect on the persistence of individual characteristics over generations? How long (in terms of generations) does the fineness of wool stay constant? Have agricultural experiments been conducted with care and precision? Are experimental findings accurately documented in stock registers with regard to variations in climate and nutrition? and Are observations on the quality of progeny characteristics accurately recorded in all aspects? “I would be obligated to write a book if I am willing to continue and try to answer these issues,” he concluded (Orel and Peaslee, 2015). They are sufficient to remind us of the complex issues that must be resolved before we can approach the truth, as “we are penetrating the innermost secrets of Nature.”

Due to his liberal beliefs, C. C. André was compelled to leave the Habsburg monarchy’s territory in 1821 and relocated to Stuttgart (see Poczai et al., 2021). He had exposed sheep breeders to study the subject of inbreeding. Festetics absorbed empirical information about animal and plant breeding. He used the word “improvement” (*Veredlung*) to refer to the process of artificial selection used to create new forms of animals and plants (Orel and Wood, 2000). He recognized that inbreeding-related malformations might have a detrimental effect on the individuals’ survival and fertility (Szabó and Poczai, 2019). Festetics substantiated his “genetic laws of Nature” *via* observation and experience in sheep, horse, goat, swine, horned cattle, and poultry breeding (Festetics, 1819a; Festetics, 1819b; Festetics, 1819c). Festetics’ rules of organic functions were tied to an organism’s fundamental life functions (*Organismus*) (see Poczai and Santiago-Blay, 2021). These are comparable to a strong constitution and excellent health, where particular “genetic laws” govern the process of heredity. Festetics’s rules established critical links between variability, adaptability, development, and inbreeding (Poczai et al., 2014). Additionally, he discussed the consequences and functions of selection in heredity, arguing that variation and inheritance are intrinsically linked in natural processes (Orel and Wood, 1998).

Another important scholar in this place and time was J. K. Nestler (1783–1841), the newly appointed professor of natural history and agriculture at the University of Olomouc (Orel, 1978). He brought the topic of scientific animal and plant breeding into his teaching in 1824, with a special emphasis on sheep breeding (Nestler 1829). He acknowledged both parents’ role in passing characteristics on to offspring, stating that “*fruitful generation with inheritance of all the significant features in the progeny is feasible only between two types (in the natural historical sense) of the same species.*” J. M. Ehrenfels, one of many breeders inspired by the work of Festetics and Nestler, said that “*environment, nutrition, and generation continue to be Nature’s levers for matter creation.*” Generation, the genetic force, is the most powerful of these three potentials when they interact (Ehrenfels, 1829; 1831).

By the mid-1830s, understanding of animal and plant breeding had advanced tremendously. More breeders were persuaded that distinct production characteristics might be inherited and enhanced via crossbreeding and selection (Orel, 1977). When E. Bartenstein, the president of the 1836 SBS, was arranging the program for the meeting, he requested J. K. Nestler to address this novel subject (Teindl et al., 1836). His reasoning was “*that the most critical issue of all, and the most urgent one at the moment, is the inheritance capability of noble stock animals.*”

Cyril Franz Napp (1792–1867), appointed abbot of the Augustinian St. Thomas Monastery at Brno in 1824, was a supporter of J. K. Nestler in his quest to comprehend inheritance. He was in charge of the monastery estates, which derived their primary income from sheep farming. Napp joined MAS within a year of his tenure and SBS 2 years later. Napp made a new and significant argument at the 1836 meeting: that the issue of heredity is related to how the interior structure of animals affects their outward forms (Orel and Wood, 1981). He emphasized the need of tackling this issue as the focus of physiological research. Napp posed the central issue at the next year’s meeting: what is inherited and how (Bartenstein et al., 1837)?

Following the 1836 meeting of SBS in Brno, Nestler published a serialized article titled, “Heredity in sheep breeding”, in which he distinguished heredity from the mystery of generation (Nestler 1837). He believed that the animal kingdom’s notions of species and race match exactly to the plant kingdom’s concepts of species and variations. “*Nature creates natural species with undeniable consistency in all species via causes beyond the control of Man.*” Humans may mimic Nature and exert control over the reproductive process, “*resulting in the creation of modified creatures that have the potential to propagate or perish in subsequent generations, depending on their inheritance*.” Nestler recognized the important contribution of abbot Napp: “*Heredity of traits from the producer (Erzeuger) to the produced (Erzeugten) relies primarily on the mutual affinity of paired animals via kinship (einseitige Wahlverwandschaft). As a consequence, a ram selected for a single ewe should match her in both inner and outward organization* (Nestler, 1837).”

Napp was articulating the “valuable research issue of heredity,” which Gregor Mendel would explain 29 years later on a scientific level. In 1843, Napp admitted Mendel to his monastery. Recognizing Mendel’s interest in natural sciences, Napp sent him to Vienna University in 1851 to study physics. Mendel returned to Brno, inspired to research the physics of plant life, with the intention of conducting plant hybridizing experiments in order to explain on a scientific level the universally applicable law of hybrid creation and growth (Mendel, 1866).

## Conclusion

In antiquity, the material foundations and methods of biological heredity, development and evolution remained a mystery. Aristotle and contemporaries accepted spontaneous generation. Their issue was not a faulty religious understanding of nature, but an inability to distinguish properly between living and non-living things. It took centuries for naturalists to demonstrate that living things exhibit unique features from non-living materials necessitating an understanding of processes rather than just decomposing events and then analyzing them. There seems to be agreement that a number of reasons contributed to the difficulty of conceptualizing hereditary principles. Although Newton’s success encouraged mechanicism, many believed action at a distance to be non-mechanistic. This devotion to mechanism drove 18th century scientists into preformationism, where they were unable to disentangle the ideas of heredity and development. For scientists in the 16th and 17th centuries, heredity was only a stage in the endless process of development. They were unaware that transmission processes can and should be researched independently. Only the unfolding process of preformed homonculi was deemed mechanical. Nobody could understand how joining two homunculi might provide a suitable beginning point for development. The problem was that although these investigators attempted to establish connections or resemblances between succeeding generations, their findings were inconsistent. Blumenbach’s force seemed to be entirely mechanical to 18th century scientists, but his “organic force” was only a word used to legitimize epigenesis.

On the other hand, French doctors did successfully disentangle inheritance from development. Since then, separating transmission from development was a critical step that both breeders and Mendel made in the 19th century. They recognized that the appearance of organisms was determined by both inherited and environmental factors. This finding sparked decades of disputes in Central Europe between naturalists, animal and plant breeders. They recognized that through inbreeding, they might increase the certainty of heredity. The term “artificial selection” was developed by C.C. André well before Darwin to demonstrate the ability of the “genetic force” to resist environmental influence through its underlying “genetic laws” as Festetics envisioned. Scientists in Mendel’s scientific society were still stumbling in the dark, speculating on the principles and laws governing the transfer of traits from generation to another. Mendel may have faced some of these obstacles, but he advanced only by disregarding them. He must have purposefully limited his analysis to elements (*Elemente der Befruchtung*) that segregate separately (Mendel, 1866). This phase ultimately resulted in the development of his theory of particulate inheritance.

## Footnotes

1 Biological heredity is defined here as the transmission and disposition of characters in organic reproduction or, in other words, the phenomenon wherein biological traits appear to be transmitted from one generation to another.

2 Those influences include: i) the prominence of data (e.g., Alexander von Humboldt’s and others’ works as well as medical data) and ii) the German idealistic philosophy, known as *Naturphilosophie* (Kaplan, 2001). Not surprisingly, in this milieu combinations of ideas and many commonly held hypotheses were rejected (Mayr, 1982).

3 More precisely, a birth is due to the conjunction of three matters: the desire for reincarnation of an orebuko (divine will), and the meeting of the woman’s blood (niñe) and the man’s sperm (ñoo, which also means water) in the womb.

4 Hippocrates and his followers were committed to the objective observation of the world. This school distinguished medicine, philosophy, and religion, which marks the beginning of medical science. Hippocrates also attributed all diseases to natural causes, omitting supernatural powers from life cycles.

5 Note that credit is given to both parents.

6 The word *tó téras* (τό τέρας) or monster used by Aristotle in *Generation of Animals* IV: 770a—770b 5–10 can also be translated as an unusual phenomenon having a negative connotation.

7 Diseases passed on from one generation to another, were referred to as *klironomikoí nósoi* (*Κληρονομικοί νόσοι*) or inherited diseases.

8 Later, with improving observation techniques, the theory of epigenesis has been proved to be correct. According to this theory, the initially undifferentiated “egg” does not contain the preformed organism, which develops only through successive steps of ontogeny.

9 This refers to a layer of embryonic epithelial tissue now called the blastoderm making up the blastula. It was easy to observe the blastoderm in chicken eggs since it is represented by a flat disc present after embryonic fertilization.

10 In humans, this miniature, preformed version of the fully-grown organism is known as the homunculus, a term also used to illustrate the contributions to sensory input and motor outputs of our cerebral cortex.

11 In fact, biologists are interested in the *Hydra* even today since the animals do not age nor appear to die at all (see Bode, 2003), but they are also considered in exploring the evolution of sleep functions (Anafi et al., 2019). The newly discovered extreme autotomy and whole-body regeneration in photosynthetic sacoglossan sea slugs fascinate contemporary biologist similary to *Hydra* (see Mitoh and Yusa, 2021).

12 Parthenogenesis is a form of natural reproduction which occurs asexually, and embryonic development takes place without fertilization. It is a quite frequent phenomenon in the living world, which can be found in invertebrates (e.g., roundworms, water fleas, scorpions, aphids, mites, parasitoid wasps, etc.) and vertebrates (e.g., fishes, amphibians, reptiles and, rarely, birds) alike. It occurs much more often in plants where it is called apomixis.

13 Polydactyly is a developmental alteration that manifests in extra digits of the hands or feet (most often in having six fingers or toes). It is caused by mutations in *HoxA* and *HoxD* gene clusters or in non-coding cis-regulatory elements (CREs). *HoxA* and *HoxD* are specific homeobox or homeotic genes, whose functioning is essential in the ontogeny of multicellular animals, thus they are also called key genes or master genes. Their primary function in embryonic development is to form body segments and related appendages such as the eyes, limbs or, in the example of polydactyly, fingers. Homeotic mutations were discovered by Edward B. Lewis in fruit flies, which earned him the Nobel Prize in Physiology and Medicine in 1995.

14 Wolff later moved to St. Petersburg, where he became the chairperson of the Academy of Sciences. He continued his work on the development and the formation of the embryo. In one of his last works before his death, he also prepared a manuscript about heredity that he based on observations of hereditary human deformities encountered on the streets of St. Petersburg. Wolff’s paper, which was only brought to the attention of others by B. E. Rajkov (1964) might require further attention in later studies of the history of genetics.

15 Monogenism considered all “races” of human beings traceable to common godly origins, in this sense dominated the pre-eighteenth century philosophical explanation of human origins (for further explanation see Goldberg, 2010).

16 The *vis essentialis* described by Wolff focused only on sustenance (nourishment), was a requisite to Blumenbach’s *Bildugnstrieb* but not by any means the *Bildungstrieb* itself. Since the *vis essentialis* existed even where there was little or no form, and also could be weak due to poor nourishment, whereas the *Bildungstrieb* remained undamaged.

17 The original phrase comes from Horace the Roman poet used in his “First Book of Letters.” Later, Immanuel Kant used it in his work “Answering the Question: What is Enlightenment (Kant, 1784).” After the publication of Kant’s work, the motto became associated with the Age of Enlightenment, translated to “Dare to know” in a bit looser translation “Dare to be wise,” or with an extended meaning “Dare to think for yourself!”.

18 In ancient Greek *génesis* (γένεσις) means origin, source, beginning, nativity, generation, production or creation, it is derived from Proto-Indo-European **ǵénh*_*1*_ *tis*, which means birth or production related to the Ancient Greek term *gígnomai* (γίγνομαι), meaning “to be produced, become or be” (Liddell and Scott, 1940; Danker, 2001).

19 See also references to antiquity in Pangenesis and the Power of Reproduction.
